# Supplementary figures and images for: An integrated approach to the characterization of immune repertoires using AIMS: An Automated Immune Molecule Separator
Source: PLoS Comput Biol. 2023 Oct 20;19(10):e1011577. doi: 10.1371/journal.pcbi.1011577 (PMC10619816; doi:10.1371/journal.pcbi.1011577)

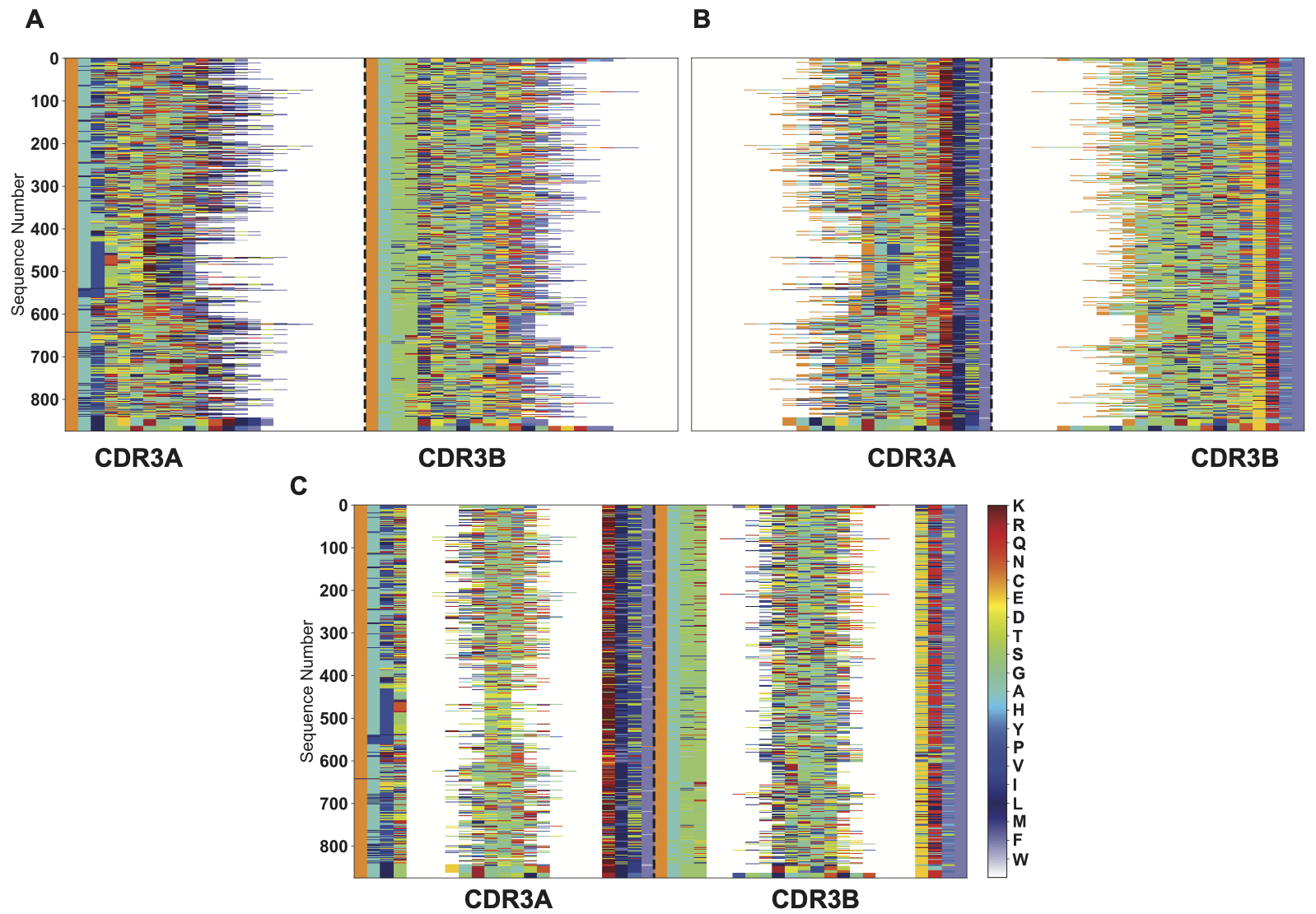

Supplement: S1 Fig — Each of these schemes are independently applied to individual key structural features by aligning to the (A) N-terminal amino acids, (B) C-terminal amino acids, or (C) “bulge” encoding as discussed in the peptide analysis described in the main text. Here the bulge padding is set to 6, i.e. 3 amino acids padding the N- and C- termini are separated for alignment, and the remaining amino acids are centrally aligned. (TIFF) [file pcbi.1011577.s001.tiff]

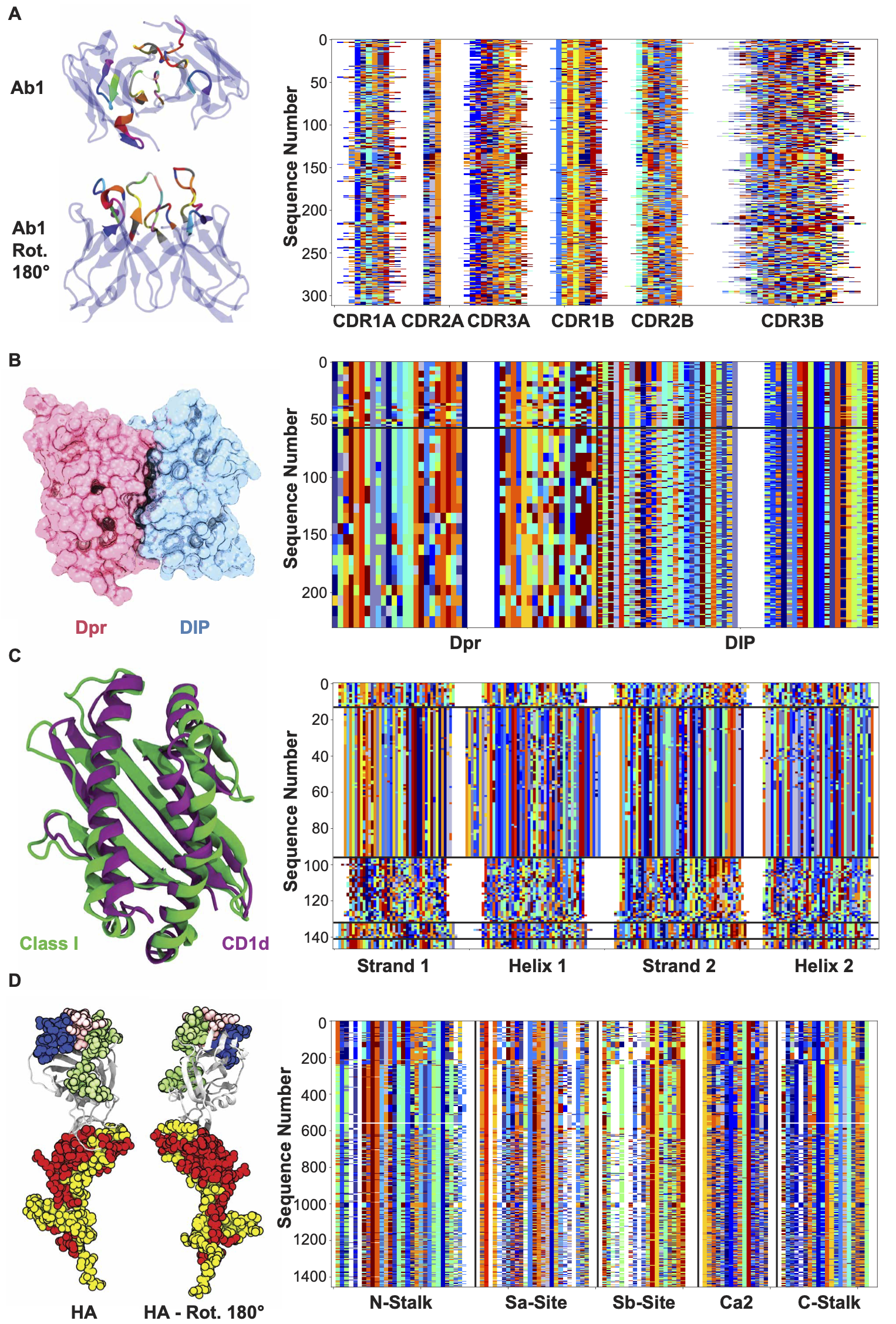

Supplement: S2 Fig — (A) Antibody encoding of all six CDR loops, structure via Borowska & Boughter et al. [68]. (B) Multiple sequence alignment encoding as discussed in Nandigrami et al. [38]. (C) MHC and MHC-like encoding of the α-helices and β-strands of these related molecules, structures via PDBs: 2XPG, 1ZT4. (D) Multiple sequence alignment encoding of Influenza hemagglutinin (HA) protein, structure via PDB: 1RUZ. Influenza MSA via 3DFlu [69]. (TIFF) [file pcbi.1011577.s002.tiff]

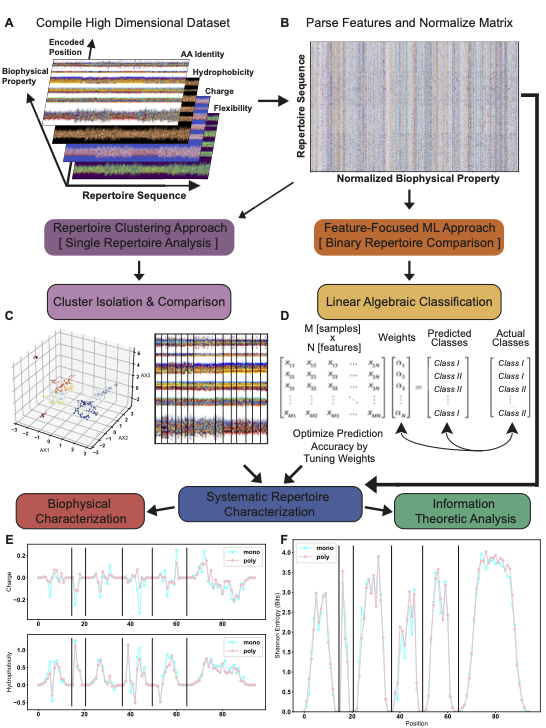

Supplement: S3 Fig — (A) Visual representation of the high-dimensional biophysical property matrix. (B) Representative parsed biophysical property matrix reshaped into two dimensions. (C) Exemplary dimensionality reduction, clustering, and re-visualization of the data in B. (D) Simplified matrix representation of the linear discriminant analysis workflow. Final repertoire characterization steps using (E) biophysical property analysis or (F) information theoretic analysis of this specific example dataset. Here and throughout AIMS outputs, “sequence position” refers to the encoded position in the AIMS alignment matrix. Vertical black lines in panels E, F, delineate core structural features (here, distinct CDR loops). All position-sensitive figures utilize the same AIMS encoding. Lines and colored boxes help guide the reader through the workflow. (TIFF) [file pcbi.1011577.s003.tiff]

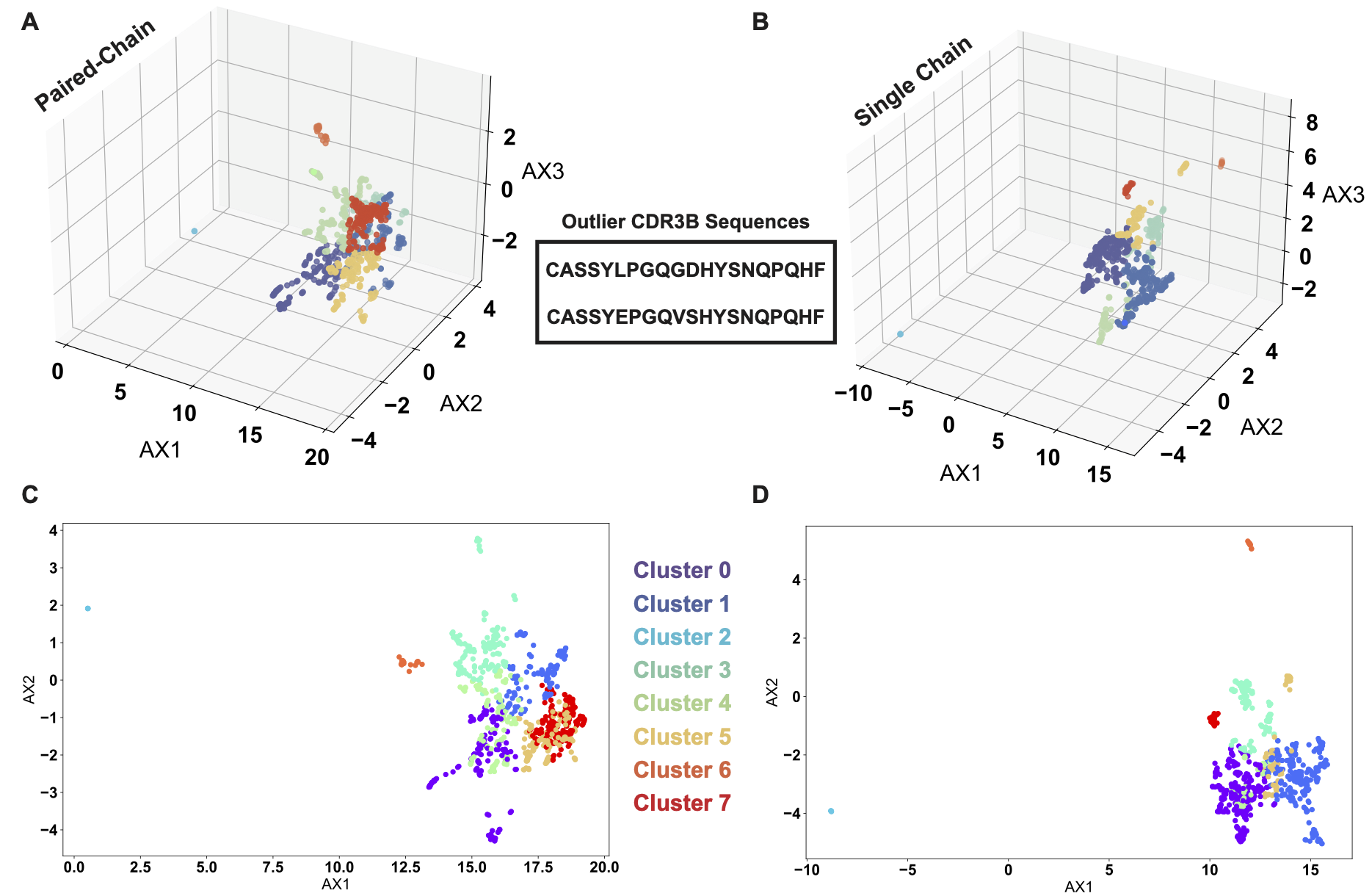

Supplement: S4 Fig — Shown are the three-dimensional clustering results for the paired chain (A) and single chain (B) data and the two-dimensional projections of these same figures for the paired chain (C) and single chain (D) data. CDR3β amino acid sequences of these outliers are highlighted in the center of the figure. Both sequences were confirmed to be the outliers in the paired chain and single-chain data. (TIFF) [file pcbi.1011577.s004.tiff]

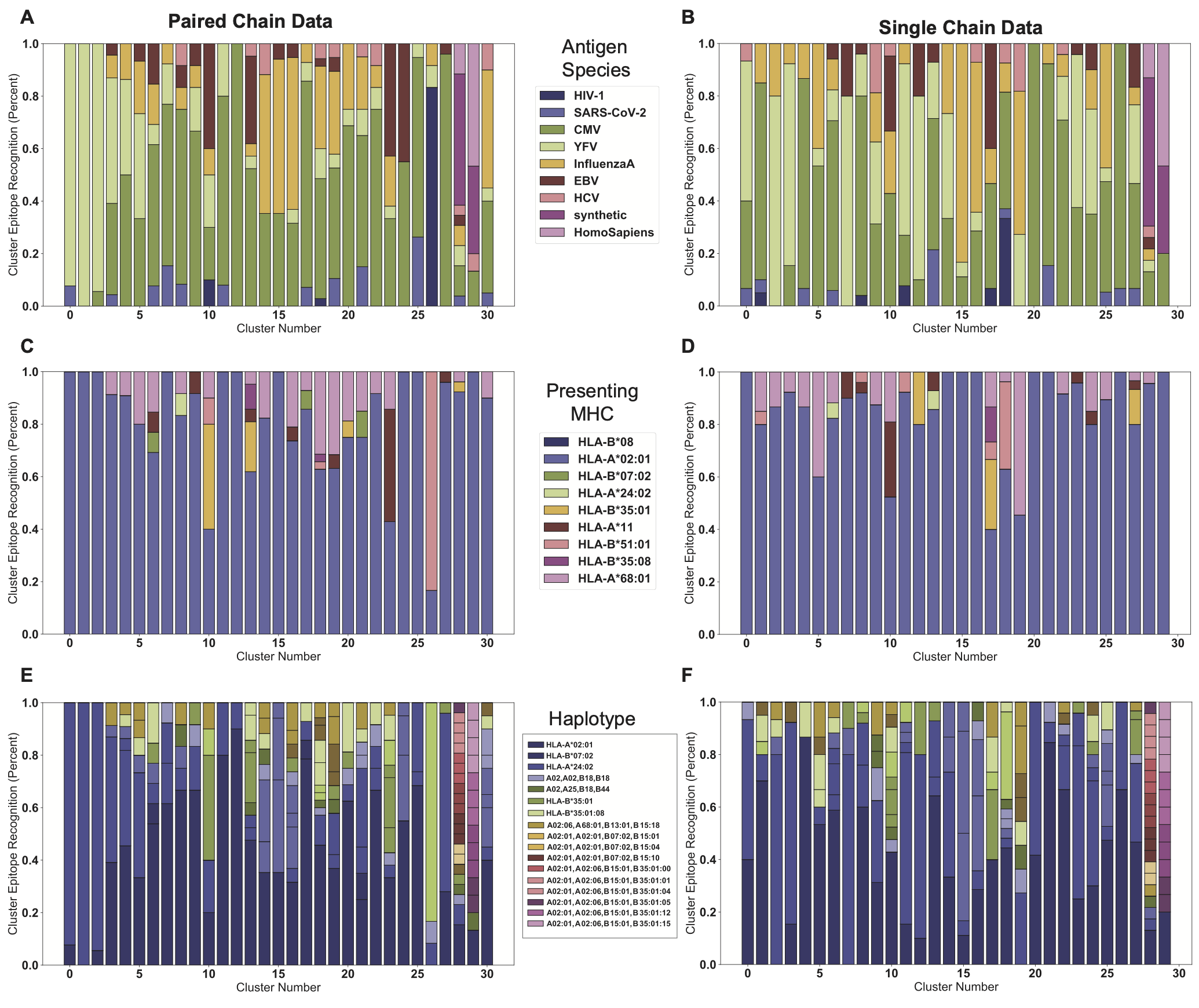

Supplement: S5 Fig — Antigen species source for each cluster member, with a cluster purity of 0.52 ± 0.26 (paired, A) and 0.39 ± 0.24 (single, B). Presenting MHC of each tested epitope for each cluster member, with a cluster purity of 0.68 ± 0.35 (paired, C) and 0.646 ± 0.37 (single, D). Organism haplotype for each cluster member, with a cluster purity of 0.46 ± 0.26 (paired, E) and 0.38 ± 0.22 (single, F). Legends are comprehensive for panels A, B, C, and D but only show a subset of the groups for panels E, F. (TIFF) [file pcbi.1011577.s005.tiff]

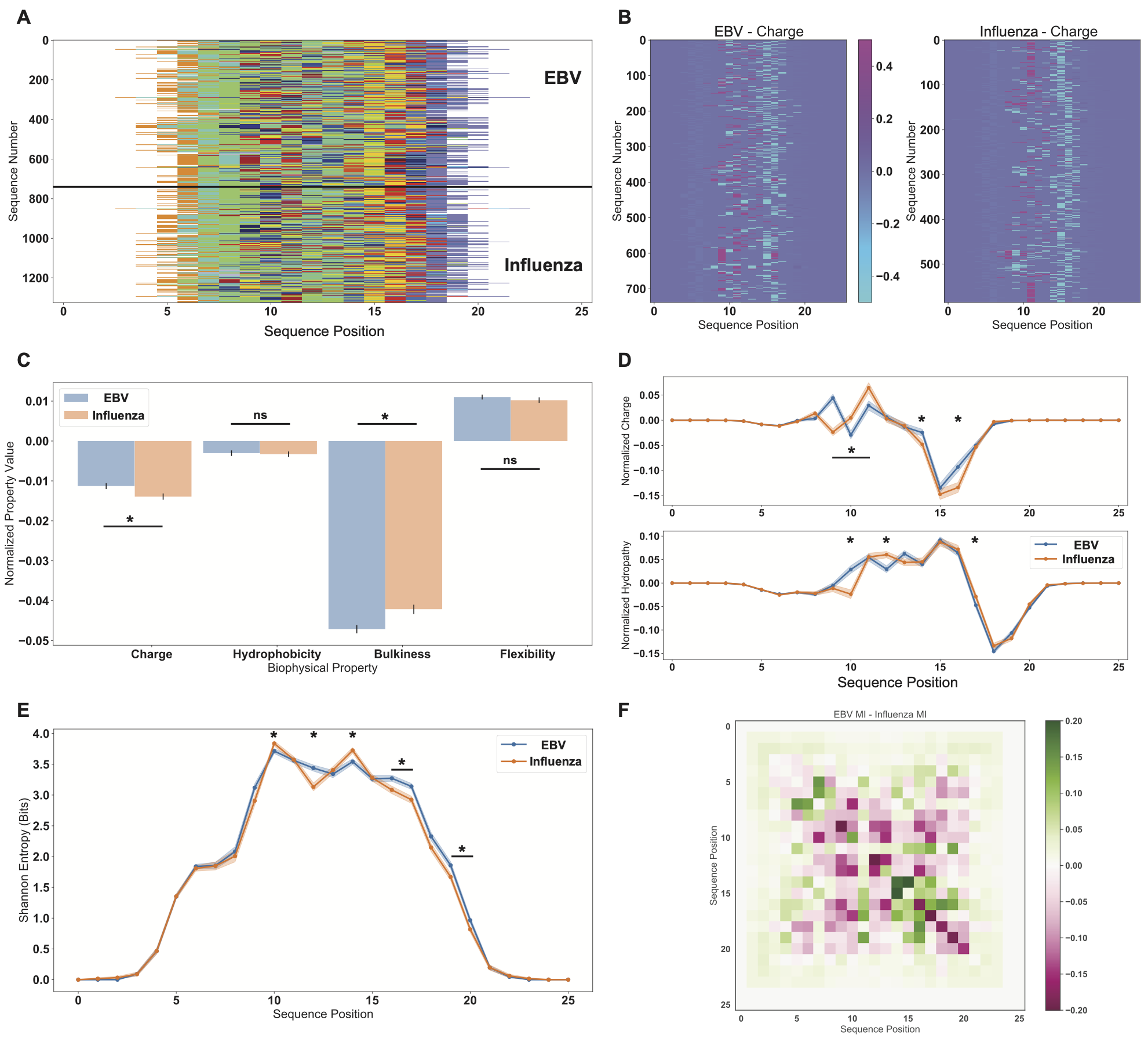

Supplement: S6 Fig — (A) Initial AIMS encoding separated by antigenic reactivity, using the central alignment scheme. (B) Example of a biophysical property mask applied to the data in (A), here specifically showing the position- and sequence-sensitive normalized charge of each sequence. (C) Net biophysical properties, i.e. averaged over all positions and all sequences, for each antigen specificity. (D) Position sensitive charge and hydropathy, i.e. averaged over the y-axis of panel B, for each antigen specificity. Information theoretic analysis concludes the characterization of an antigen-specific repertoire, with the position sensitive entropy (E) and mutual information difference (F). Statistical significance of differences between these two populations are calculated for panels C, D, and E using a non-parametric permutation test (Methods). Averages and standard deviations in panels C, D, and E calculated using a bootstrapping procedure (Methods), with standard deviation in panels D and E represented as a shaded region about the solid line averages. ns—not significant, *—p < 0.05, solid bar with * above—contiguous region of p < 0.05. (TIFF) [file pcbi.1011577.s006.tiff]

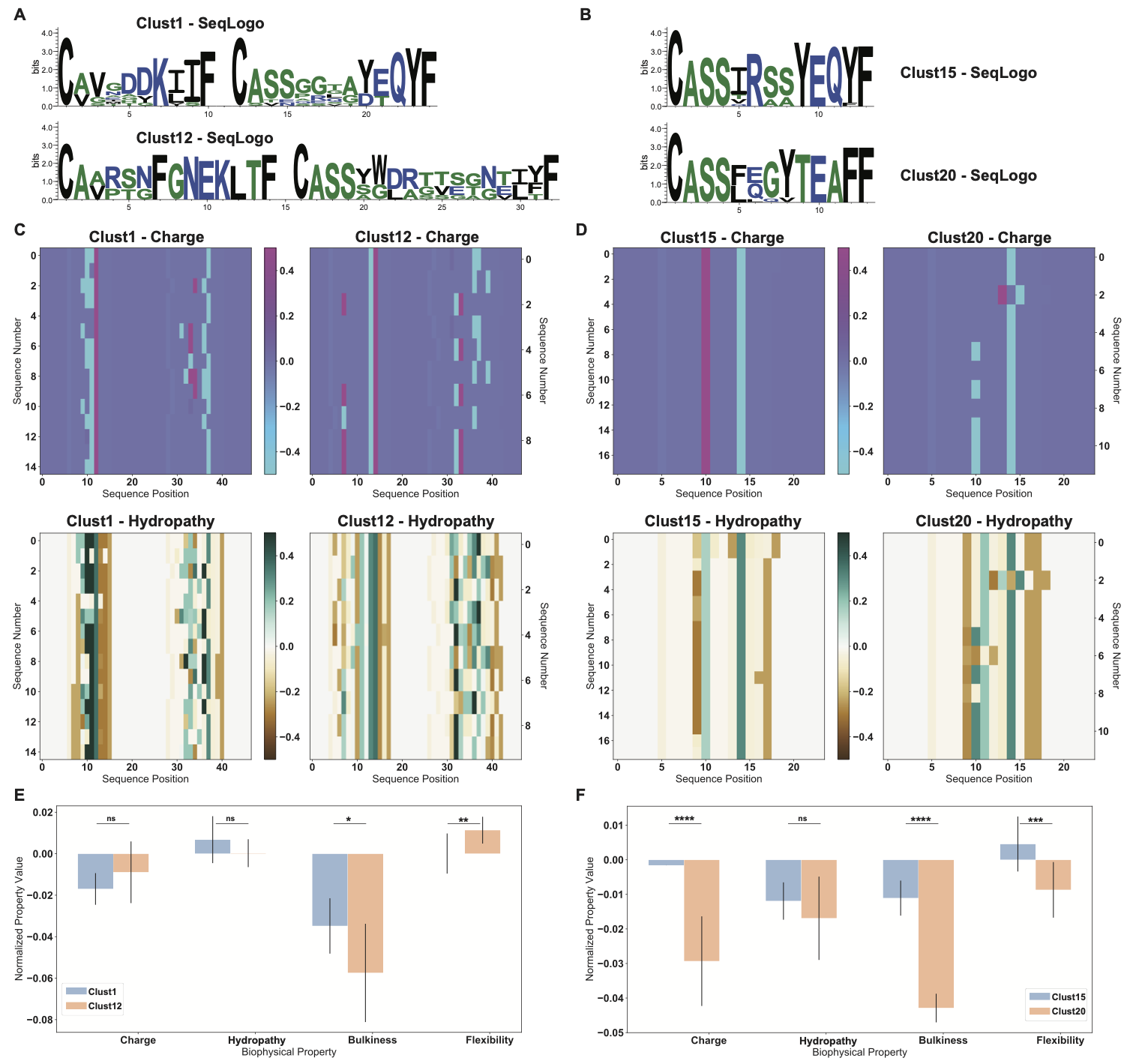

Supplement: S7 Fig — (A, B) Sequence logos of the selected clusters of Fig 3A and 3B, as generated by WebLogo [70]. (C, D) Biophysical property masks of charge (top) and hydropathy (bottom) for each cluster of sequences. The position-sensitive biophysical property masks of Fig 3C and 3D are generated by averaging over the y-axis of these plots. (E, F) Net averaged biophysical properties, i.e. averages over the x- and y-axes of panels C and D, of four out of the sixty-one available AIMS properties for each cluster of sequences. Statistical significance of differences between these two populations are calculated for panels E and F using a non-parametric permutation test (Methods). *—p < 0.05, **—p < 0.025, ***—p < 0.01, ****—p < 0.001. (TIFF) [file pcbi.1011577.s007.tiff]

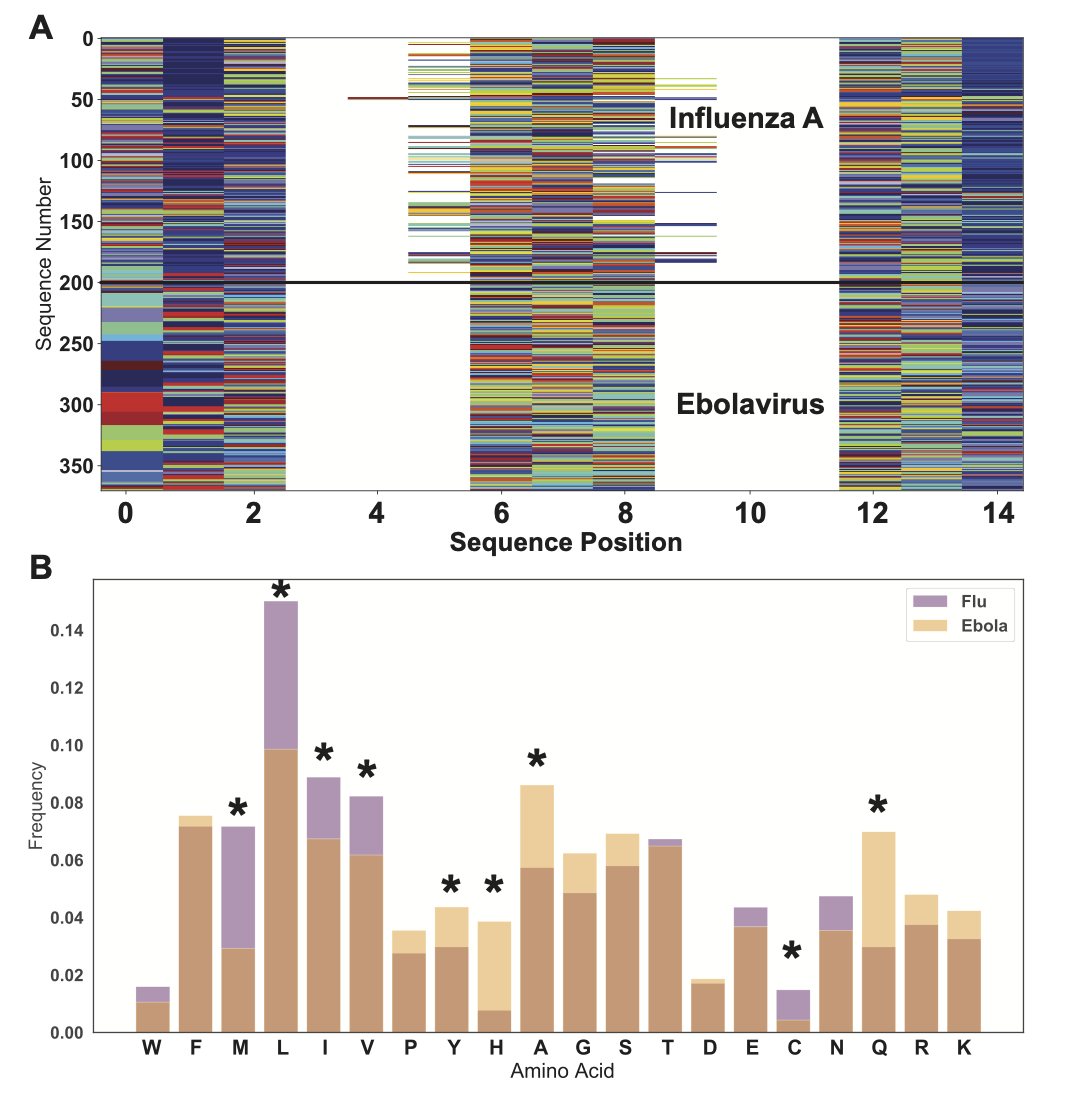

Supplement: S8 Fig — (A) AIMS-encoding using the bulge alignment scheme of the dataset of Influenza A and Ebolavirus derived peptides. (B) Position-independent amino acid frequencies for the HLA-A2 presented Influenza A peptides and the HLA-B15 Ebolavirus peptides. Statistical significance of differences between these two populations are calculated for panel B using a non-parametric permutation test (Methods). *—p < 0.05. (TIFF) [file pcbi.1011577.s008.tiff]

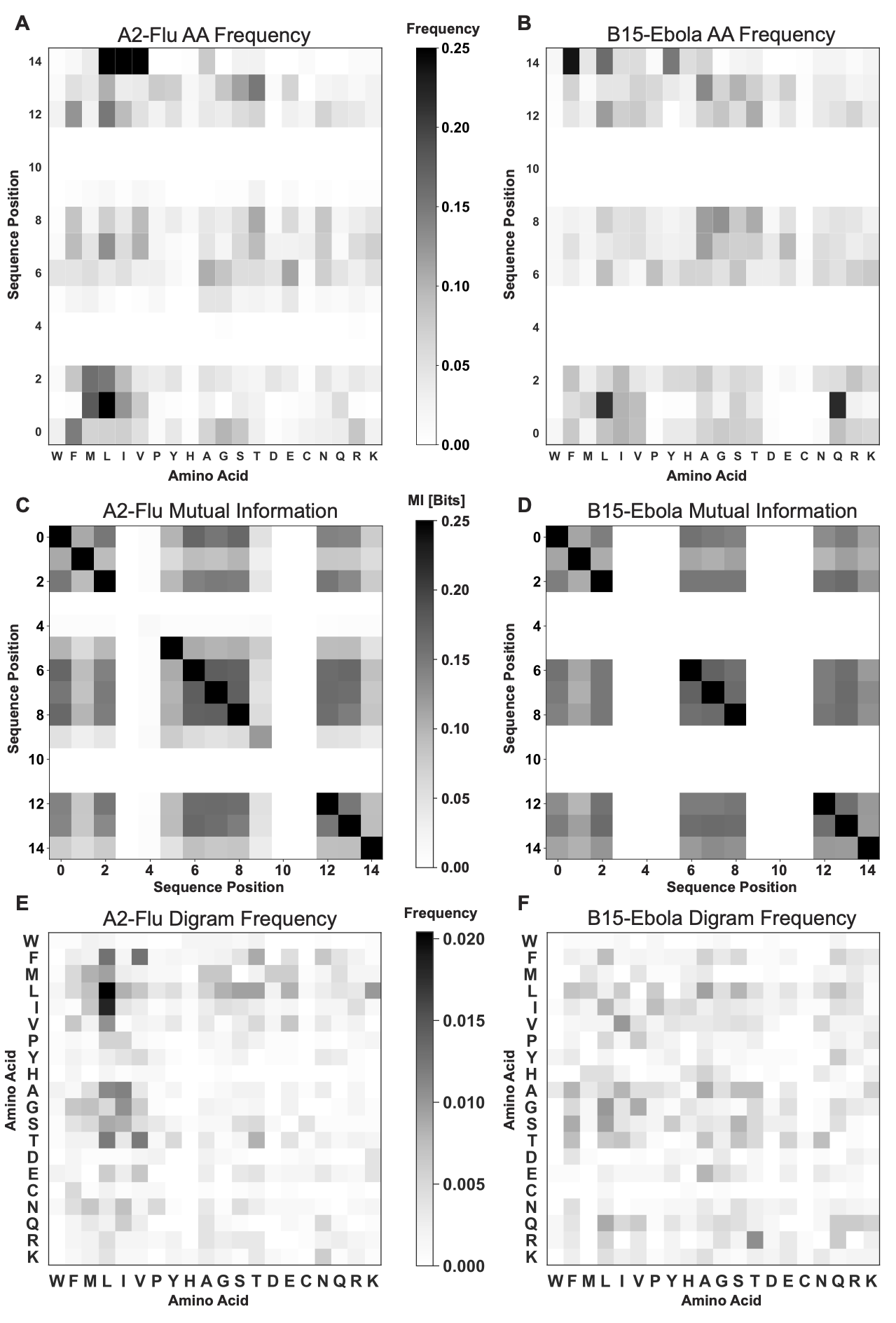

Supplement: S9 Fig — Position-sensitive amino acid probability distributions for (A) Influenza A and (B) Ebolavirus derived peptides. Position-sensitive mutual information calculated between each encoded sequence position for (C) Influenza A and (D) Ebolavirus derived peptides. Amino acid digram frequencies for (E) Influenza A and (F) Ebolavirus derived peptides. (TIFF) [file pcbi.1011577.s009.tiff]

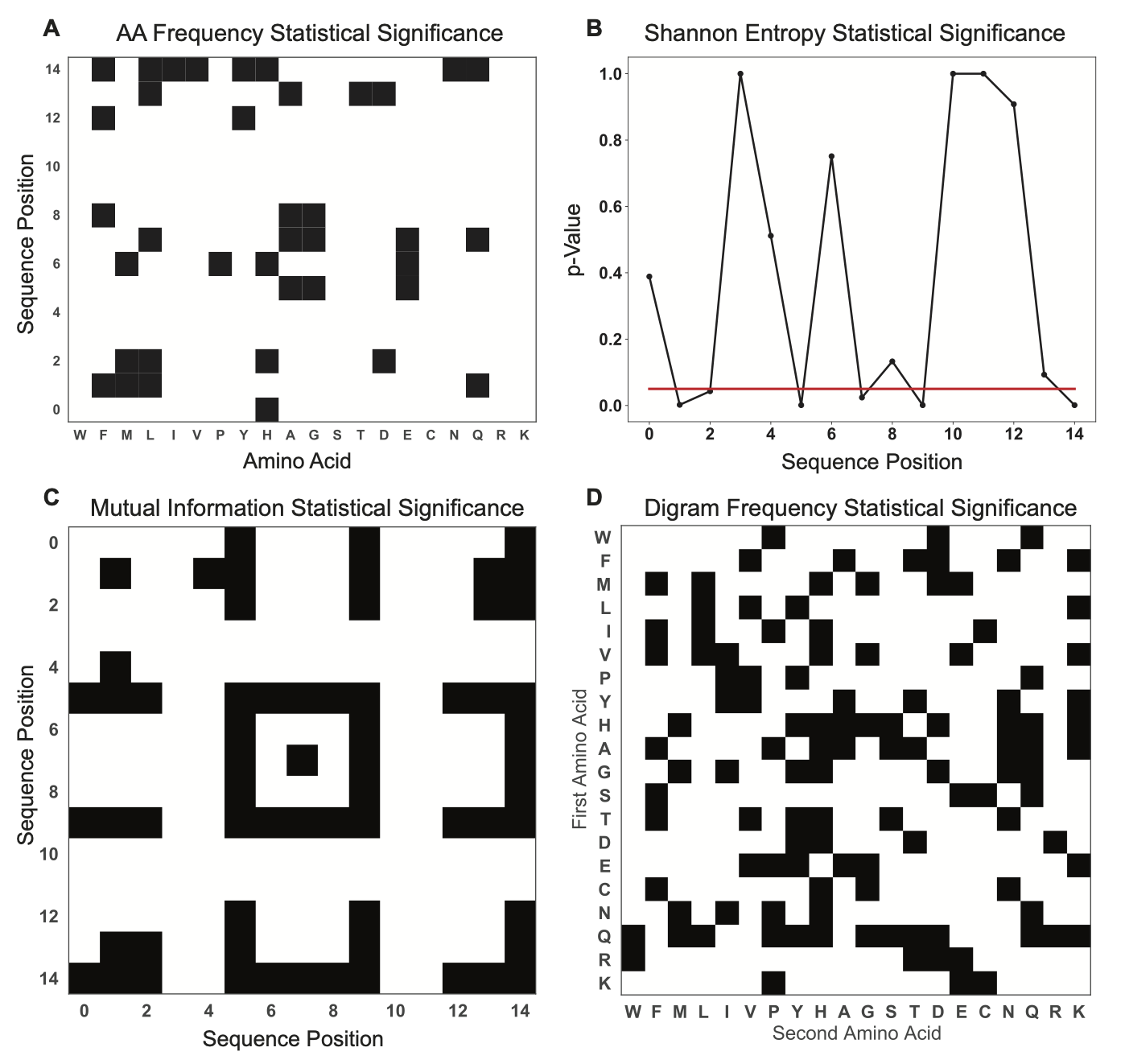

Supplement: S10 Fig — Statistical significance is shown for the amino acid frequency difference (A), the average Shannon entropy difference (B), the mutual information difference (C), and the digram frequency difference (D). Statistical significance of differences between these two populations are calculated using a non-parametric permutation test (Methods). For all tests, a threshold of p < 0.05 is used, denoted by either the solid red line in panel B or the presence of a filled (black) square in the matrices of panels A, C, and D. (TIFF) [file pcbi.1011577.s010.tiff]

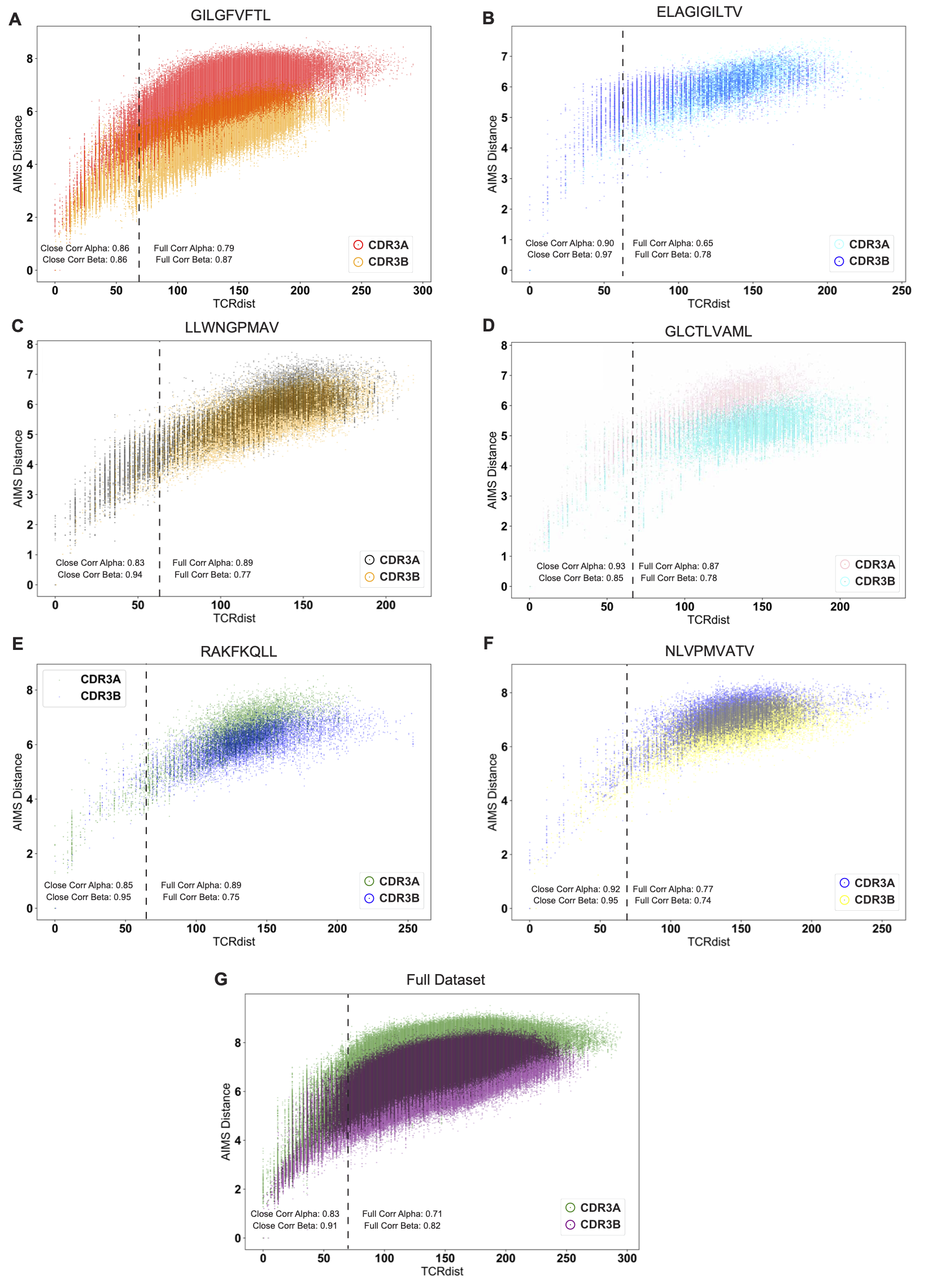

Supplement: S11 Fig — Here only the sequence distances calculated via TCRdist and AIMS between full-CDR sequences of Mayer-Blackwell et al. [56] are compared directly for the TCRα- and β-chains. Correlation coefficients between these distance metrics are reported for the full set of sequences and for closely related sequences, which are delineated by the dashed vertical lines at a TCRdist of 60 units. TCRs are isolated from human T cells in response to each antigen listed above the plots (A-F) or for the full dataset of TCRs (G). (TIFF) [file pcbi.1011577.s011.tiff]

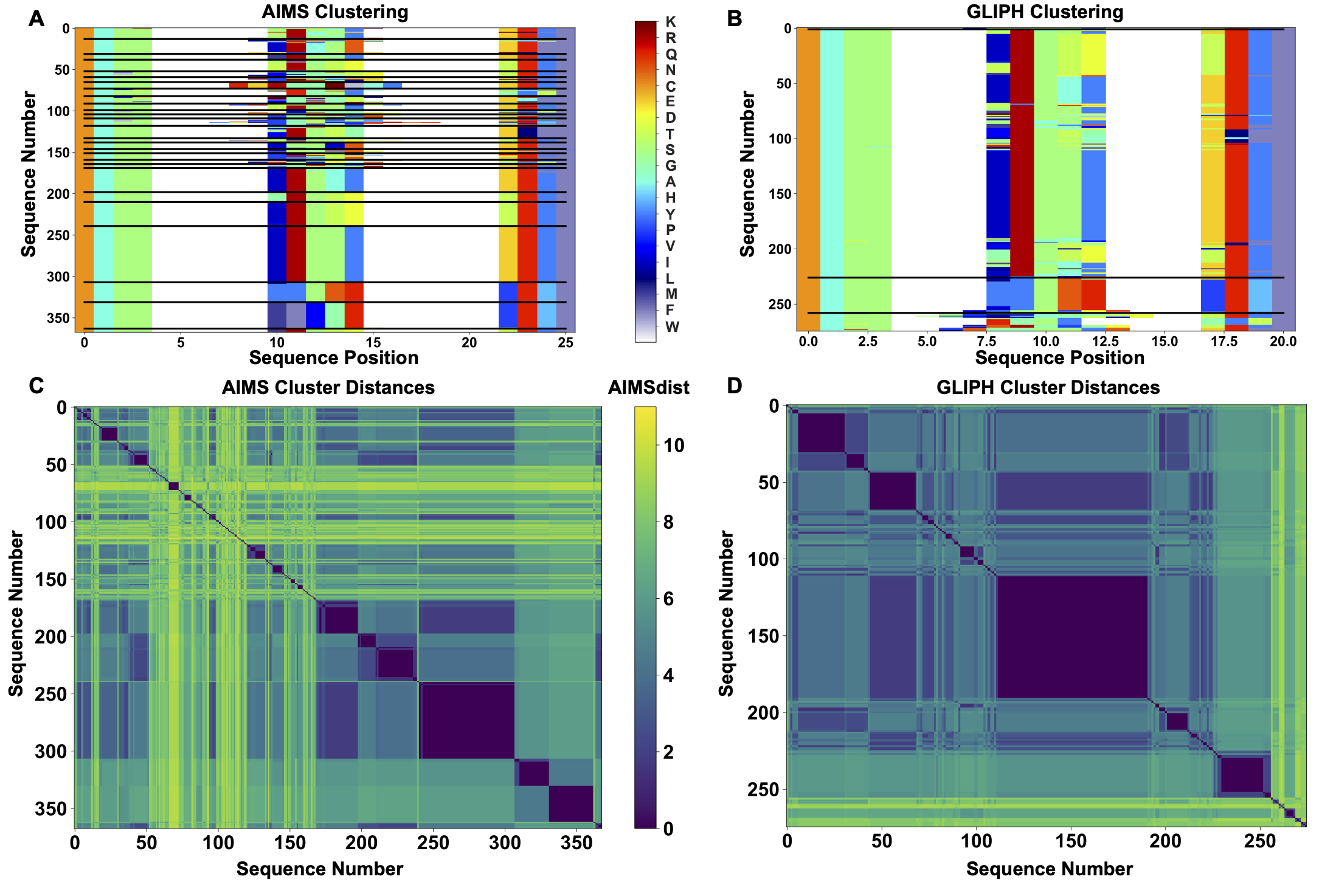

Supplement: S12 Fig — The AIMS clusters (A) are generated from the curated Influenza A reactive sequences from Glanville et al. Supplementary Table 1 [26] subject to a UMAP projection and DBSCAN clustering (eps = 0.15) of the biophysical property matrix. while the GLIPH clusters (B) are taken directly from Glanville et al. Supplementary Table 7 [26]. Calculating the AIMS distances between the sequences within the AIMS clustering (C) or the GLIPH clustering (D) shows highly similar patterns for the most similar sequences, suggesting both methods are capable of identifying highly pure clusters, albeit with a higher resolution in AIMS. AIMS additionally identifies highly biophysically distinct yet self-similar clusters (sequences 50–150) compared to the previously identified specificity groups. (TIFF) [file pcbi.1011577.s012.tiff]

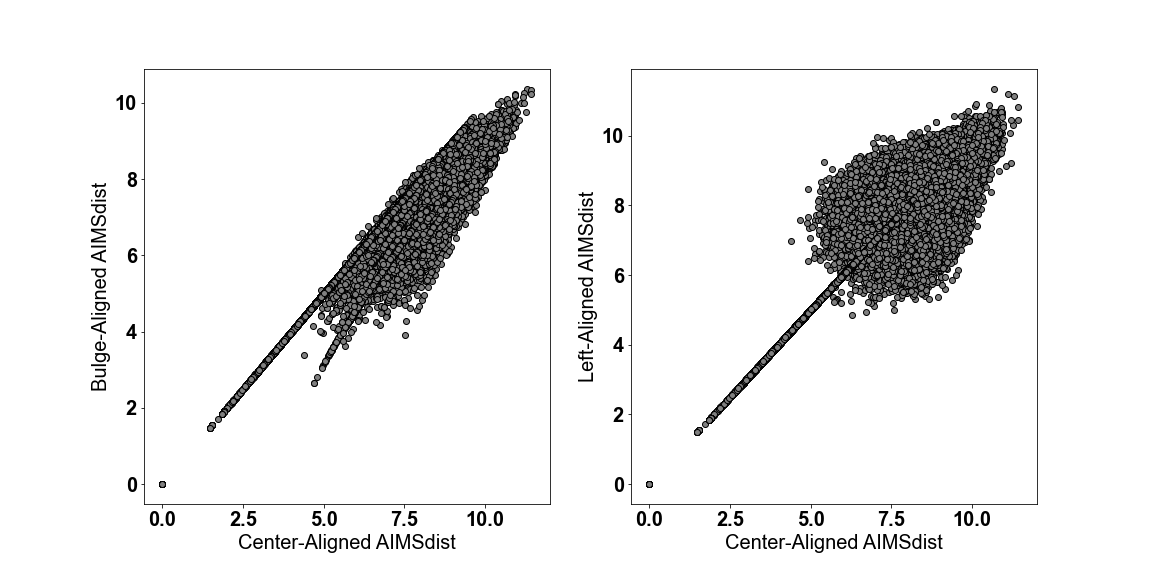

Supplement: S13 Fig — We see that largely the distances are preserved for similar TCRs, yet there is some divergence in the calculated metric at higher distances for comparisons between the bulge- and center-alignments (left) and the left- and center-alignments (right). (TIFF) [file pcbi.1011577.s013.tiff]
