# Supplementary material for: An integrated approach to the characterization of immune repertoires using AIMS: An Automated Immune Molecule Separator
Source: PLoS Comput Biol. 2023 Oct 20;19(10):e1011577. doi: 10.1371/journal.pcbi.1011577 (PMC10619816; doi:10.1371/journal.pcbi.1011577)
Supplement: Attachment — Submitted filename: PLOS_Review_responses.pdf [file pcbi.1011577.s017.pdf]

Response to PLOS Comp Bio Reviews – July 1st, 2023

We thank the reviewers and the PLOS Comp Bio editorial team for their time and effort spent towards providing a fair and useful critique of this manuscript. Here we provide a point-by-point response to the reviews, and an updated version of the manuscript. Throughout this response, text from the editor or reviewers will be italicized and displayed in gray font, while the authors responses will be in non-italicized black font, and excerpts from the text are in italicized black font. Line numbers refer to the PLOS-formatted version of the manuscript, not the preprint-formatted version.

Reviewer #1: *The authors present an encoding scheme for a more comprehensive analysis of immune repertoires.*

However, in the current form, this manuscript doesn't make much sense. The text is a random assortment of statements with motivation or research question and the figures are difficult to make sense of.

I like the idea of a new encoding scheme for immune receptor data that enables novel and more unbiased analyses. However, in the current form, I don't see or understand how the authors achieve this goal.

I suggest the authors rewrite their manuscript with more focus and clarity. Also, the manuscript seems to be TCR-heavy, a bit more space on the antibody side would be nice.

We thank Reviewer 1 for their comments. To address the concerns over the readability of the text and the figure legends, we have made changes throughout the entirety of the manuscript, and clarified figures where necessary.

We hope that, in concert with the changes suggested by reviewer 2, the newest version of the manuscript is much improved.

Regarding the focus on TCR and peptide analysis, this was an intentional bias in the text. The software was initially developed for the analysis of antibody sequences [Boughter et al. *eLife* 2020, PMID: 33169668]. The current manuscript focuses on 1. The expansion of the software to include a range of other molecular species, including non-immune molecules and 2. The formalization of the analytical pipeline that users can follow. The manuscript should be thought of as a companion piece for the use of the analysis software and as an outline of its various capabilities such that other researchers can compare their own approaches with ours.

Reviewer #2: *This paper provides a useful and important perspective for the sequence analysis of TCRs and antigens presented on MHC. The core of the approach is encoding methodology for sequences. This encoding couples a centered alignment (TCRs) or an anchored + centered alignment (pMHC) along with a high dimensional biophysical representation of each amino acid. Furthermore, the authors have put an*

impressive effort in making their code accessible, even building a GUI interface – this is commendable in a field that often assumes technical knowhow and rarely puts a premium on usability.

We thank you for your appreciation of the GUI. Its creation required a lot of effort, and we hope it does improve the usability.

The authors show the usefulness of their method in three main ways:
1) Characterization of biophysical properties of collections of sequences (Figs 2, 4, S5, S6)
2) Clustering of similar sequences (Figs 3, 6, S3, S4)
3) Probabilistic and Information theoretic characterization of collections of sequences (Figs 2, 5, S5, S7)

The characterization of biophysical properties is of particular interest and would be a useful tool if the following points can be addressed.

Major points:
1) The core of AIMS is the encoding of sequences, of which alignment is a crucial part. It would be very helpful if the authors quantitatively compared different alignment schemes. While there are some qualitative explanations for the centered vs bulge alignment schemes, there is no quantitative comparison of downstream analyses (many of which are entirely conditional on position in the alignment) between these strategies and other alignment methods. In particular, how does a standard multi-alignment, or a combination left-right alignment perform?

We agree with the reviewer that the appropriate choice of alignment is important for the analyses offered by the AIMS software. The differences in these alignment strategies will depend strongly upon the underlying data. However, we can estimate how alignment differences might manifest themselves using the same AIMS distance metric as Figure 7 (Supplemental Figure S12). We see from this supplemental figure that for similar TCR sequences, the alignment assumption does not alter the distance in biophysical property space. However, at higher AIMS distances, there can be deviations, although the TCRs are still classified as “distant”.

Additionally, throughout the text, we frequently refer to “user preference”, and this is a situation where user input is key. In some TCR use cases, users may want to focus on TRAV/TRBV or TRAJ/TRBJ gene-usage, and so a left, right, or bulge alignment strategy would be preferred. In others, the more variable regions (center of CDR3) are most important, so a central alignment scheme may be used. As mentioned in the text (lines107-110) users are encouraged to test multiple alignment strategies to ensure robust results.

To explicitly address the impact of these assumptions, we have now added a paragraph in the discussion section addressing the exploratory nature of the AIMS software (Lines 514-527):

It is important to note that the analytical tools provided by AIMS are best utilized both as a means of identifying differences between datasets and as an exploratory tool. Many of the decisions made at each stage of the analysis can alter the downstream interpretations, so users are encouraged to test different alignments, projection methods, clustering algorithms, and clustering options. While choices in alignment do not strongly alter the underlying structure of the input dataset (Fig. S12), they can generate differential emphasis on distinct features. Further, as seen in Supplemental Figure S3, the inclusion or exclusion of certain sequences can distort the projected spaces used for sequence clustering. Thorough investigation should include multiple iterations of analysis, testing how single or paired chain data alter outputs, and how inclusion of mixed or single antigenic specificities in a given AIMS run can provide new and exciting insights. Users are encouraged to use the graphical user interface or add their own code to make AIMS an indispensable tool for data exploration, hypothesis testing, and figure generation all in one easy-to-use package.

Also, why is the bulge alignment method used for the TCR sequences in Figure 6 (comparison with GLIPH).

The bulge alignment was used for the TCR sequences in Figure 6 to highlight that such an alignment is a viable way to analyze TCR sequences as well as peptides. As mentioned in the above response, the bulge alignment strategy when applied to TCR sequences allows for an increased emphasis on the V- and J-gene encoded regions of the TCR.

We can show that for this specific application, alignment did not strongly alter the clustering results. Using the central alignment scheme, to match other TCR analysis in the manuscript, we see how similar the results are in the figure below:

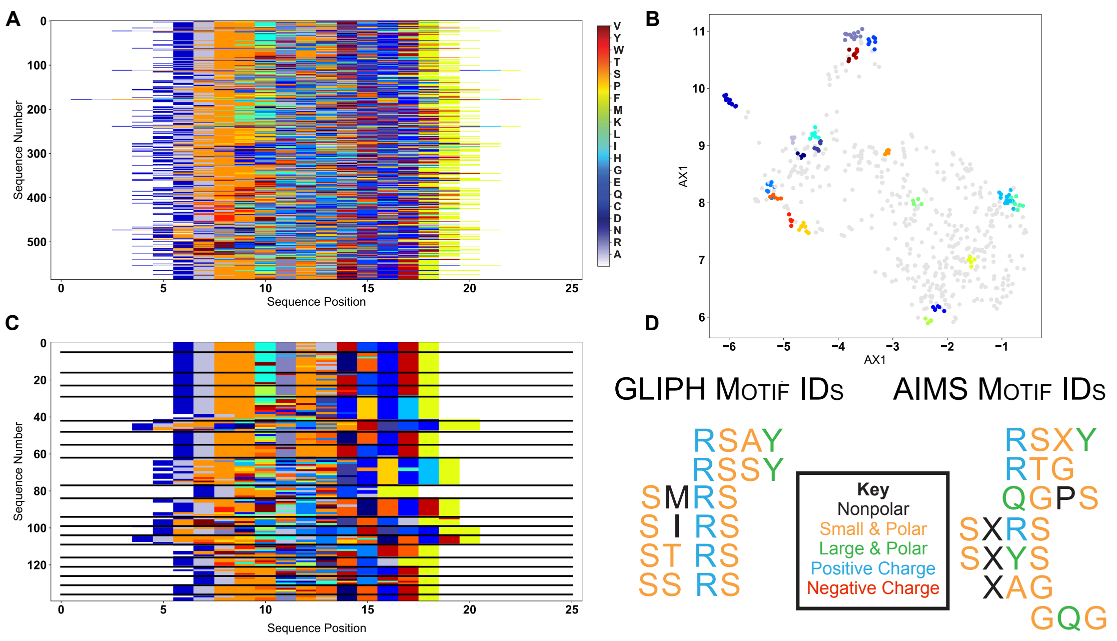

While the clustering data looks different, we are still largely able to identify the most prominent, biophysically similar sequences. Qualitatively, panel C above looks similar to Figure 6C. However, we can calculate the AIMS distance between the sequences in the clusters identified using either the center- or bulge-alignment strategies to quantitatively assess this similarity. We can see from Review Figure 2 that the first 40 bulge-aligned and the first 60 center-aligned sequences are biophysically similar according to their AIMS distance. These contiguous stretches of biophysically similar sequences suggest the clustering identifies the same sequences, regardless of the alignment method used. Here the color bar gives the distance between the bulge-aligned and center-aligned sequences in each cluster.

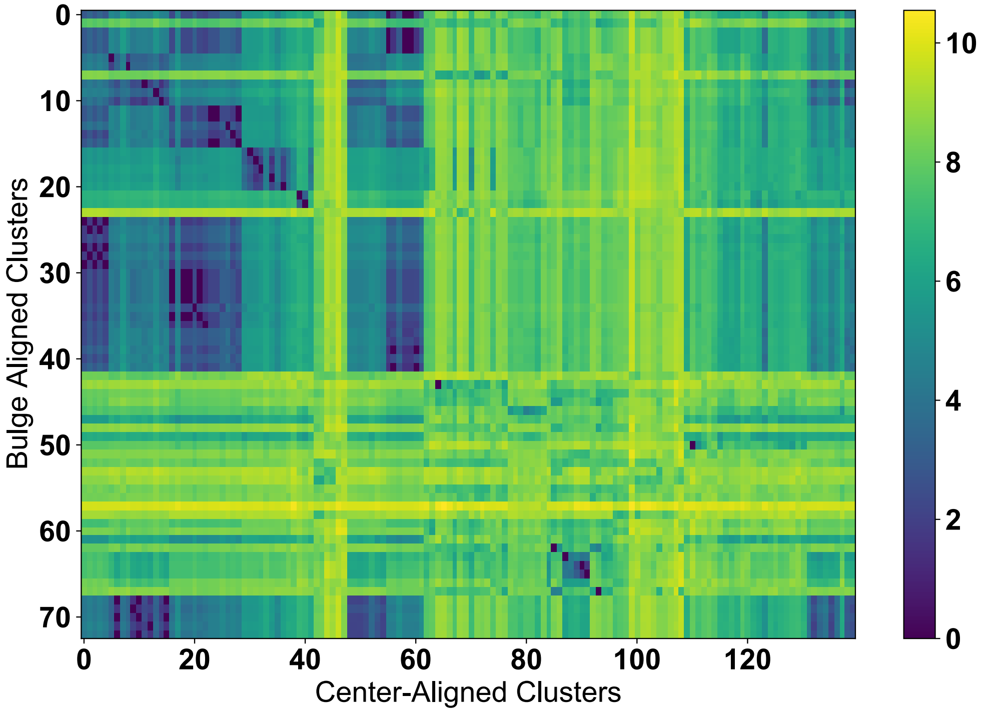

2) The authors don't include discussion of statistical significance or sample size effects throughout the paper. The authors mention that there is a built in tool in AIMS to do bootstrap estimation of errors – they should include this statistical analysis wherever they can. I highlight a few areas in subsequent comments.

We thank the reviewer for emphasizing what has long been a shortcoming of the AIMS analysis. Statistical analysis has been historically difficult due to almost all of the data in the AIMS analysis being non-normally distributed. However, as the reviewer suggests, such statistics are crucial for AIMS to become a robust analytical technique.

As such, we have added functionality across all AIMS modules to optionally output statistics. This largely comes in the form of the permutation test to deal with the non-normally distributed data and bootstrapping to generate standard deviations for plots.

From the first AIMS bootstrap error estimation modules, we have generated a faster approach calculating the AIMS biophysical property matrix in an early step in the analysis. This makes the permutation test and bootstrapping procedures more computationally tractable for most users, although it may still be slow for larger datasets.

Statistics have been added to Figures 4, 5, S5, S6, and S7. Other Figures involve either descriptive labels of individual sequences or are schematic in nature, making application of statistical measures inappropriate. A discussion of these statistical tests can be found in the methods section.

3) The alignment strategies used by AIMS means that the coverage of different positions varies. This is never explicitly shown and has both noise effects and direct finite sample size effects on some quantities. For example, the estimate of entropy is capped at log(N) where N is the number of points.

We apologize for not having given a clear derivation of the upper bound of the entropy. The position-sensitive entropy is capped at $\log_2(N)$, but where N is the number of possible entries at a given position. So, for AIMS encoding, this means the entropy is capped at $\log_2(21) = 4.39$ Bits, where N=21 the number of amino acids +1 (+1 being for the padded zeros added to the encoding).

You can clearly see the entropy going to 0 in areas that have low coverage. I highlight areas where this is an issue, but plots of the alignment position coverage should be included (Figs 2, 4, 5, S5) and a note made about why the entropy goes to 0 in places. The authors may also want to consider using a normalized entropy.

The entropy goes to 0 largely when there are gaps in the alignment filled by padded zeros (as between the CDR loops of TCRs). However, as the reviewer notes, there are also regions where the entropy converges towards zero largely due to sparse sequence coverage. These regions also interestingly have artificially *increased* entropy per the central alignment strategy. The V- and J-gene regions of TCRs are relatively well conserved, and the central alignment strategy creates mismatches in these well conserved regions, boosting the entropy.

We can quantify the extent of how alignment alters entropy using the GLIPH TCR dataset as an example (Review Figure 3, below). We see from this figure that in this case, the differential alignment indeed boosts the entropy in the conserved V-gene region (left) and that the structure of the entropy as a function of position is largely conserved outside of this region. It is important to note that the alterations to the entropy as a function of position will vary with the datasets of interest, but are most evident in applications of antibody and TCR analysis where length is more variable.

Due to the high conservation in the V- and J-gene encoded regions, and the fact that all the information contained in this region can be summarized by gene usage plots, we typically prefer to minimize the importance given to these regions in favor of the center of the TCRs. While there is clearly an effect on the calculated entropy, we accept this tradeoff due to the ability of other methods to regain this information. This point has been expanded upon in the discussion.

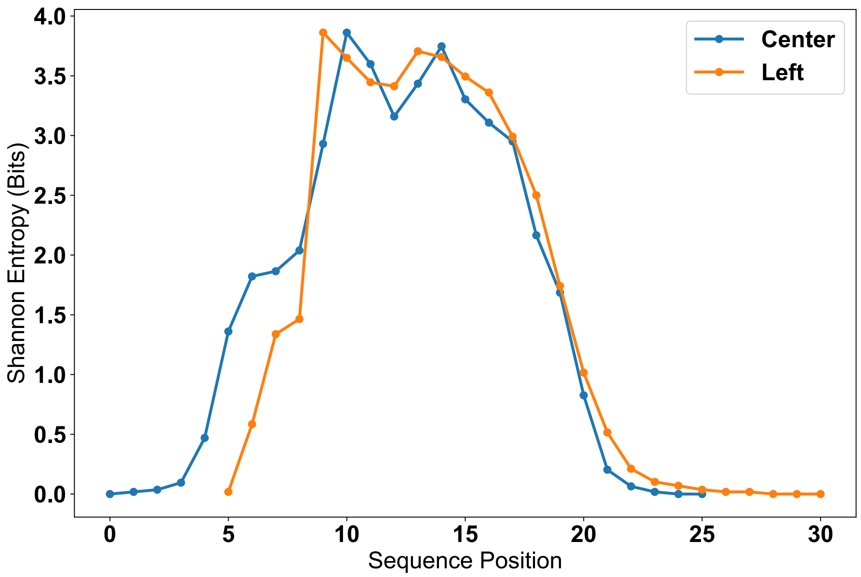

4) For the clustering module, there is no discussion or analysis on the repeatability/stability of the clustering. TCR repertoire samples can vary wildly in size over a few orders of magnitude, so a sense of how sample size dependent the clustering is and how stable it is to subsampling would be extremely helpful.

We thank the reviewer for bringing up this point. Reproducibility in clustering is indeed a big issue, so much so that the python UMAP module we utilize has a section dedicated solely to this issue [https://umap-learn.readthedocs.io/en/latest/reproducibility.html]. While this issue is discussed in depth both within the code and in the AIMS ReadTheDocs page [https://aims-doc.readthedocs.io/en/latest/AIMS_cluster.html], we have now added a more explicit discussion of these effects in the manuscript [Lines 514-527, included in this response, above]

Sample size likely is not as big of an issue as sample heterogeneity. As can be seen in Supplemental Figure S3, a single biophysically distinct cluster of TCRs can significantly alter the structure of the projected space. This is why AIMS should typically be considered an exploratory tool for the data. Much like clustering of single cell RNAseq data, the onus is on the user to heed the warnings of the developers. To further enforce this point, we have added a new section to the AIMS ReadTheDocs homepage:

A Note on Exploration with AIMS

The AIMS software should be considered as both a means for exploratory searches through data to generate hypotheses and as a tool for rigorous quantification of differences between molecular subsets. In the former application, users can freely explore their data, tuning different AIMS parameters and seeing how these changes alter identified clusters or comparison groups. However, in the latter application, users should carefully record the setting of each tuned parameter. Analysis using AIMS should be considered akin to modern RNAseq analysis, where the rigor of a given analytical tool depends on proper implementation by the user. Reproducibility is key!

5) Figure 5. It would be useful to show the probability distributions, mutual information, and di-grams for the two peptide clusters individually before you show the difference between them. As mentioned in major point #3, using normalized entropy will highlight the dip in entropy due to the conserved anchors. Lastly, there are finite sample size effects on the mutual information (e.g. see Treves and Panzeri https://www.tandfonline.com/doi/abs/10.1080/0954898X.1996.11978656). The authors should check the magnitude of these effects as it is not clear that any of these differences plotted are significant or noise.

We agree with the reviewer that the raw probability distributions and mutual information calculations will add much needed context when considering the differences in these metrics between the two studied populations. These plots have been added to the supplement (Supplemental Figure S8) along with calculations of statistical significance for these differences (Supplemental Figure S9).

Regarding the use of normalized entropy, the current, standard entropy calculation appears capable of capturing the dip in entropy due to the conserved anchors. As for the significance of the differences in the mutual information, new calculations show large regions of statistically significant differences in the mutual information. However, these considerations of finite sample size effects are being considered for the newest versions of the mutual information calculations.

6) The authors do not include any results or plots for the machine learning/LDA classifier techniques. Either such an analysis should be added or the section from lines 378-394 should be cut from the results section and the application discussed in the discussion. Similarly, the section in the methods for LDA should be cut unless the analysis is used in the paper.

The reviewer is correct. The LDA section was only included to let users know AIMS supports such machine learning approaches. However, due to the already substantial length of the manuscript, we would prefer to not go in-depth discussing these approaches. The LDA section will be removed from the results but expanded in the discussion as a potential application.

7) Comparison to GLIPH. The authors have an excellent methodology for characterizing the effectiveness of their own clustering. They should do a comparison between GLIPH clusters and their own using cluster purity (or other quantity – see major point #2) in order to see which method performs better.

Using the AIMSdist feature, which was created for direct comparisons to TCRdist, we can calculate biophysical similarity of sequences in either AIMS clusters or GLIPH clusters for the Influenza dataset (Supplemental Figure S11). We can also utilize inter-cluster distance to estimate how each method identifies biophysically distinct clusters. Using Supplementary Table 7 of Glanville et al. and comparing directly to the AIMS clustering of raw sequences of Supplementary Table 1 of Glanville et al., we find the AIMS clustering matches and even somewhat outperforms the capabilities of GLIPH clustering.

First, we note that the same sequences GLIPH identifies as distinct are likewise identified by AIMS. AIMS, however, goes a step further, providing an improved resolution distinguishing between sequences that are grouped into a single cluster by GLIPH. We see these similarities in the distance matrices, yet equally striking are the differences between the two matrices. AIMS identifies a full 100 additional sequences that are strongly biophysically divergent from the identified GLIPH sequences (as quantified by AIMSdist). These clusters maintain strong self-similarity, suggesting that AIMS surpasses GLIPH by identifying smaller, potentially significant, biophysical outliers still capable of identifying specific antigenic targets.

8) Details on how some quantities and plots (e.g. cluster purity, how amino acid gaps are factored into biophysical averages) are missing from the methods. Similarly, any parameter choices for the alignments and clustering should be included.

We agree with the reviewer and have significantly altered the methods section, now including more precise parameter choices and generalities to the AIMS analysis, rather than extended details. The old methods section is now included as an appendix.

Minor points:

1) The authors stress the importance of not conditioning the analysis on sequence length, however much of the biophysical and information theoretic analyses performed are done on clusters of sequences with almost identical sequence lengths (e.g. TCR clusters in Fig 4, HLA presented peptides in Fig 5). Does AIMS cluster sequences of similar lengths together? Is the analysis of position dependent biophysical properties dependent on similar length sequences?

This is an important, nuanced point regarding the AIMS analysis, and again the answer is conditional on the type of analysis used. If users are dealing with a heterogenous dataset, like from PBMCs, which bear TCRs capable of recognizing any number of pathogenic targets, and a stringent clustering technique like DBSCAN or OPTICS is used, then AIMS clusters are likely to include only sequences of similar length.

However, if more homogenous datasets are used, like those from tetramer sorting, where TCRs with singular specificities are isolated, we can expect that even these stringent clustering methods will be more likely to cluster sequences of varying lengths, due to convergences in recognition strategies and “motifs”. Regardless of the data used, a broader clustering algorithm like KMeans will certainly cluster sequences of differential length that lie in proximity in the projected space due to likewise broad biophysical similarities.

2) The authors do not concretely translate what “Position” is on the x- axis for Figs 2E and 2F. It should be made clear if this is position in the aligned TCR sequence space, and furthermore what the overall coverage of sequences per position is (see major point #3). The authors should also explain what the vertical black lines are. It would also be useful to briefly state what the example repertoire being plotted is.

We thank the reviewer for bringing these issues with the figure legends to our attention. More descriptive figure legends have been included where appropriate and a description of certain novel AIMS features have been added to the methods. These include figure captions 2, 3, 4, and 5.

*3) The authors use a nice readout of cluster purity to assess the effectiveness of their clustering. This is a very nice analysis, but the quantity is sensitive to the overall distribution of the categories. This is a bit of a problem as there is a significant skew in the datasets used (e.g. HLA-A*02 is far more studied than other HLA and comprise a large fraction of the total peptides). While not essential, the authors may want to use a quantity that isn’t so sensitive to this skew (e.g. an entropy based calculation or even just mutual information).*

We thank the reviewer for the suggestion, and will continue to improve the AIMS software using these comments as a guideline. It is important to note that as with any analytical tool, users must consider the appropriateness of the analysis they are carrying out.

However, thanks to this reviewer’s comments, we can help users quantitatively assess this appropriateness using statistical arguments. The statistical tests used to address comment 2 of this review can also be used to test for statistically significant enrichment of certain populations in a given cluster, giving a quantitative test for this skewing. Now changing our cluster purity metric to display cluster fraction of each metadata annotation (in this case peptide specificity) we can then assess whether this fraction is statistically significantly enriched ($p < 0.05$) over background. The results of these cluster purity calculations for the data in Figure 3E can be seen below, where the color bar of the right panel gives the p-value for each enriched population in the left panel:

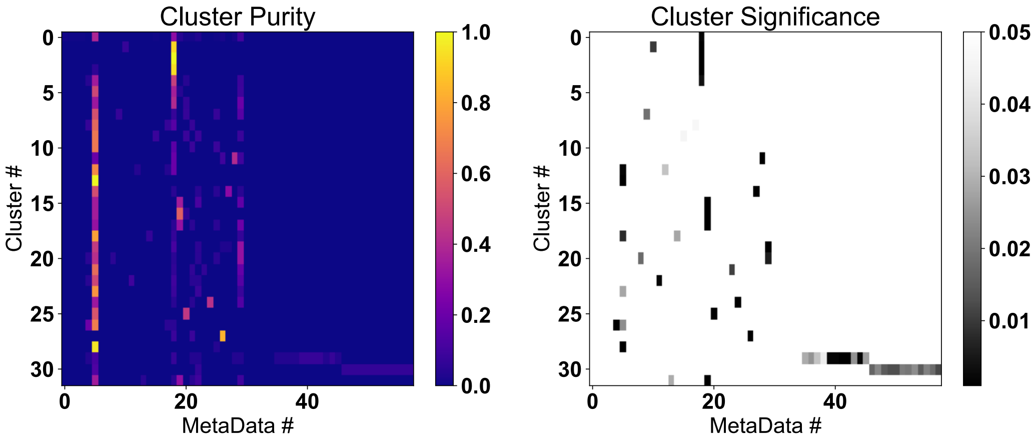

4) The alignment of MHC class I presented antigens assumes a central bulge. While this is a good assumption for most human HLA some human HLA and mouse MHC have central anchor positions (for example: https://www.ncbi.nlm.nih.gov/pmc/articles/PMC2248166/). Is this bulge model applicable in these circumstances? Is a more flexible approach possible to align the sequences to account for the variability in anchor positions?

The strength of the bulge alignment scheme is that absent a literal bulging of long peptides, it is still capable of providing a more physiologically relevant alignment of the peptides. Unlike the case of TCR or antibody analysis, the N- and C- termini of peptides are hugely important carriers of information, and as such should not be subjected to the averaging effect of the central alignment scheme. The bulge alignment allows for this information to be preserved while also maintaining attention on the likely TCR-contacting regions in the center of the peptide.

Regarding an anchor-based alignment, this is certainly a possibility, but something that could introduce improper assumptions. The strength of the AIMS analysis is a relaxation of rigid assumptions like explicit structural prediction or pairwise interactions. While anchor regions do strongly bias peptide presentation, the identification of peptide anchors appear to be more like guidelines than actual rules. There are multiple cases of crystallized structures with unfavorable anchor residues [Nguyen et al. *Biochemical Society Transactions* 2021 PMID: 34581761] and the introduction of stronger anchors to neoantigenic peptides can negatively affect TCR binding and T cell recognition [Smith et al. *PNAS* 2021 PMID: 33468649]. As such, we believe keeping these simplified assumptions, rather than generating potentially incorrect assumptions about anchor identities, should make AIMS a more robust analytical tool.

5) In the clustering module (lines 237-241), the authors speculate on why TCR 1A, a tumor-isolated TCR, does not cluster. Speculating over a single TCR sequence is a bit of a stretch especially as they do not highlight what specific biophysical property of this TCR makes it an outlier.

In our opinion, the notable aspect of this TCR 1A is not that it is a biophysical outlier, but rather that it is more biophysically similar to "background" TCRs in the dataset. Densely clustered TCRs that are outliers from other TCR populations are what are included in AIMS clusters. Non-notable TCRs, like 1A, are often left unclustered. But the reviewer is right that the considerations we added about TCR 1A are not very strong, and we have removed them from the revised text.

6) Fig 4 would be improved by taking coverage into account (see major point #2). I would also recommend aligning the sequence representations in A and B to the position axes in C and D. It would be helpful to the reader to identify the clusters as being specific to the NLVPMVATV antigen or LLWNGPMAV antigen instead of the legends "Clust1" vs "Clust12". Also, the plots are a bit confusing to follow. Can the authors plot the mean +/- std? At the least choosing colors that are more distinguishable than pink and purple would be helpful -- the highly transparent plots are hard to tell apart.

We thank the reviewer for the suggestion to align the data in panels A and B with those of C and D, we think this greatly improves the interpretation of the figure. We make the other associated changes, but note that a raw standard deviation is improper for panels C and D, as the data are not normally distributed. We instead include a bootstrapped standard deviation and report significance using the permutation test. These nonparametric methods allow for statistical inferences to be made from these non-normally distributed data.

7) Lines 370-373. It would be useful if the authors had a methodology for determining significance of N-gram motifs, but this may be beyond the scope of this paper.

We have included statistical significance of these N-gram motifs in Supplemental Figure S9, and find that many of these N-grams are indeed significantly different ($p < 0.05$), between the two datasets. However, if the reviewer meant "significance" in the colloquial sense, arbitrarily extending N-gram analysis (say, to a 9-gram) will eventually find statistically significant differences between datasets in almost every test case. We have now included a section in the text warning readers and users of AIMS of this issue in the fundamental use of N-gram analysis (Lines 358-364):

In addition to the standard analysis pipeline outlined here, the analysis can be extended arbitrarily to include N-gram motifs, providing the potential to identify regions with a propensity for certain tri-grams or higher-order motifs. Care must be taken when utilizing these N-gram formulations, however, as extension to the extreme such as in the analysis of nine-gram motifs for peptide datasets will identify statistically significant but not particularly meaningful data.

8) Lines 373-377. This section is written in a confusing manner. It may clarify things for the reader if the authors specified that entropy is additive when the distributions are independent, so the summation provides an upper bound (as they state).

Given the reviewer's comment and rereading these lines, we now believe this discussion of the additive nature of entropy does not add much to this section of the text. We have removed these lines in favor of the above discussion of N-gram significance.

9) The methods around the AIMS software reads like a README. A more concise methods that provides the precise parameter choices and algorithms used to generate the results of the paper would be preferred.

Per the suggestion of Reviewer Comment 8 above, we have substantially reorganized the methods section. Hopefully the Methods are now more appropriate.
