# Supplementary material for: An integrated approach to the characterization of immune repertoires using AIMS: An Automated Immune Molecule Separator
Source: PLoS Comput Biol. 2023 Oct 20;19(10):e1011577. doi: 10.1371/journal.pcbi.1011577 (PMC10619816; doi:10.1371/journal.pcbi.1011577)
Supplement: Attachment — Submitted filename: PLOS_Rev_response2_final.pdf [file pcbi.1011577.s018.pdf]

Response to PLOS Comp Bio Reviewers – Revision 2 09/03/23

We thank the reviewers and the PLOS Comp Bio editorial team for their time and effort spent towards providing a fair and useful critique of this manuscript. Here we provide a point-by-point response to the reviews, and an updated version of the manuscript. Throughout this response, text from the editor or reviewers will be italicized and displayed in gray font, while the authors responses will be in non-italicized black font, and excerpts from the text are in italicized black font.

From the editor: In light of the reviews (below this email), we unfortunately cannot proceed to publication at this time, but invite the resubmission of a significantly-revised version that takes into account the reviewers' comments. Specifically, the reviewers have raised concerns regarding scientific novelty with regards to the prior publication of the AIMS framework, and the lack of clear novel scientific results, which would need to be addressed in a revised manuscript.

We thank the editor for their handling of the manuscript, and hope that the accompanying revisions improve the manuscript. We note to the editor regarding their concern as to the relative novelty of this work that the AIMS software presented in the current manuscript is vastly different from that presented in the first AIMS-related publication [Boughter et al. *eLife* 2020]. The incorporation of non-antibody data types, the clustering and subsequent isolation of these clusters for analysis, metadata integration, and a wide range of additional features outlined in this manuscript were not present in the 2020 publication.

Reviewer #1: The authors have addressed all of my concerns.

We appreciate the reviewer's comments on this manuscript, and hope that this revision remains satisfactory.

Reviewer #2: The authors have substantially improved the content of the paper. In particular, the addition of statistical considerations in almost all of the previously flagged areas highlights where the AIMS may be most useful. There are a few (mostly minor!) outstanding issues listed below:

We thank reviewer 2 for their previous comments on the manuscript and are glad we were able to address the majority of their concerns.

Content revisions:

1) The authors have still not adequately addressed the finite sample size issues for the information theory quantities. While the authors have accounted for the upper bound of the entropy due to the state-space having 21 possible entries, they still do not account explicitly for the varying coverage due to alignment. The maximum entropy for a particular position will also be capped by the coverage: S_max <= log(max(N, 21)) where N is the number of sequences that align to the position without the padding. It is

crucial to include a plot alignment coverage by position so that the finite sampling can be accounted for: as the authors note, if a different alignment scheme is used the coverage is different and the resulting entropy by position plots change substantially.

We agree with Reviewer 2 that the finite sample size is an important issue. We have now added functionality to AIMS that explicitly warns users when sequence coverage in a specific region may be low. The coverage metric we use is calculated as N_AA/N at each site of the encoding, where N_AA is the number of sequences where a gap is not present, and N is the total number of sequences. We have added this coverage metric to Figure 5B (now 4B), and let readers know in the figure legend that this coverage applies to all position-sensitive metrics in the figure (Panels A, C).

An example of this is Figure 5. While I cannot be sure of the particulars without a coverage plot, the lack of alignment to positions 3-5 and 9-11 is the dominant features of figs 5B and 5C. Furthermore, the coverage differences between these peptide pools probably accounts for some of the most visually obvious features of the plot. Based on the plots I would guess that the HLA-A*02 Flu peptide pool includes more peptides longer than 9 amino acids so the alignment more often includes positions 5 and 9 which appear to never be aligned to by the HLA-B15 Ebola peptide pool (again, without a coverage plot this is impossible for me to determine). If a reader is not aware of these coverage artifacts they will likely be focused on that instead of the likely more biologically relevant entropy reduction at the anchor residues.

We appreciate the reviewer giving an example of where this coverage metric would be particularly useful. We agree that this metric is critical for proper interpretation of the figures, and as mentioned above, have added it to Figure 5 (now 4). Some of the differences in the figure are, in fact, due largely to differences in coverage, and without the benefit of seeing the distribution of peptides (Supplemental Figure 8A) in the same figure, these coverage differences are non-obvious. We appreciate the reviewer's improvement of this figure and the AIMS analysis as a whole.

I would suggest the following actions to rectify this:
a) At a minimum, coverage plots of the alignment of each peptide pool should be included in the Supplementary figures
b) The finite sample size effects of entropy could be largely dealt with by only analyzing positions for which the coverage is sufficiently high (i.e. >20). Similarly it is probably wise to restrict the position-dependent amino acid probability distributions to positions with sufficient coverage.

In addition to addressing the reviewer's concerns by adding the coverage plots suggested by point (a), we have also added an explicit warning in the software (not included in the manuscript) when coverage is low (point b). This exceedingly low coverage warning is printed as an output in the notebook code, and will be incorporated into the GUI in the near future.

2) If the authors have decided to not include any LDA analysis the discussion of LDA

applications should be restricted to the discussion, not the results section (see paragraph from lines 167-180) or a main figure.

In line with reviewer 2's stylistic comment below, we have removed this section entirely from the text. The associated figure has been moved to the supplement.

3) Figure 4 is much improved! The addition of the regions of significance is a great addition. I would recommend making sure the yticks in fig 4A are integers. For C/D) I still suggest adding an xlabel, but the figure alignments with A/B do alleviate the main concern.

We thank the reviewer for their previous comments that helped to improve this figure. As reviewer 3 also pointed out, there was a mistake and a rough-draft version of the new Figure 4 (now 3) was accidentally uploaded to the resubmission. The suggested corrections have been made.

4) While GLIPH is normally used as a qualitative motif tool, I would just reiterate that I think the authors are missing an opportunity for a quantitative comparison of the clustering functionality. This may not be essential for publication, but the authors have an opportunity to demonstrate to potential users any advantages they have over GLIPH groupings.

While we believe that the direct comparison of AIMS distances between GLIPH clusters and AIMS clusters is likely a fair comparison between the two (Supplemental Figure S12), we have now added a quantitative comparison using simulated data as suggested by Reviewer 4 (Supplemental Table S2). GLIPH performs much worse than both AIMS and TCRdist, clustering only 28% of the receptors, with single sequences belonging to multiple clusters. However, the use of simulated data may be an unfair test case for GLIPH, as it was explicitly generated for the identification of expanded motifs. The developers of GLIPH would likely not recommend using GLIPH with simulated or naïve datasets. However, this is also a benefit of our approach that there are no such restrictions to the analysis.

Style concerns:

While I understand that the authors want to highlight the flexibility and motivation of each element of the AIMS tool, the result is that this paper is long, wordy, and reads like a README with the authors addressing the "AIMS user" instead of the reader. I flag this not as a concern for publication, but instead for the potential reach of AIMS as a tool. I think the utility of the tool would be better highlighted if the text was half the length; with some of the exploratory/"user options" cut; and more of a focus on what can be learned from the results. The authors don't want to miss out on potential users of their tool because readers cannot get through the paper.

We appreciate the stylistic comment, and agree that initial drafts of the manuscript were a bit verbose. We have cut over 3 pages of text and one figure from the initial draft of the manuscript and have made an effort to make the writing more concise.

Reviewer #3: This is a description of the AIMS software package, meant to complement the original publication and present some new features. The manuscript is clearly written. There don't seem to be any specific novel biological insights; the manuscript is more a series of usage examples. Here are a few specific comments and suggestions.

Line 112: "the flanking regions of bound peptides are 'buried' as highly conserved anchor residues that bind to the MHC platform and are unable to contact TCR " -- this is not true for peptide position 1, which points outward and can be contacted by the TCR and serve as a specificity determinant (talking about MHC class I; see e.g. PDB 5jzi).

We appreciate the reviewer pointing out this oversimplification in the text. We have altered the text to highlight that not all peptides are contacted in these central regions:

For class I MHC, the flanking regions of bound peptides are frequently 'buried' as highly conserved anchor residues that bind to the MHC platform (Fig. 1A,B). The majority of TCR contacts are made with the central regions of the peptides that bulge out of the MHC binding groove in the case of longer peptides [Tynan2005]. However, exceptions to this paradigm may not be uncommon, with TCRs capable of contacting the often-buried peptide N-terminal residue [Wang2017] and the C-terminal residue potentially extending out of the MHC pocket [Guillaume2018].

Could the amino acid colors in Fig.1 be made more consistent with biophysical similarity, rather than just based on alphabetical ordering? Following some of the previous sequence-logo type color schemes, for example, where positively charged amino acids are blue, negatively charged are red, polar are green, nonpolar gray (or variants of these colors). It might make the alignments more visually interpretable. It doesn't make sense to me that K and M are right next to each other (and A and R), for example, or that K and R are so far away. A similar argument could be made for ordering the amino acids by similarity, in figure 5A for example, rather than alphabetically. This might make it easier to see trends in preferences. Something like: WYFMLIVAPGCSTNQDEHRK

We agree that the suggested changes may help with interpretability of the alignment figures. We believe small tweaks like these can create large improvements for the users of AIMS. We have now incorporated an option for users to define custom amino acid orderings for all plots used in the manuscript. For this manuscript, we opted for a slightly modified amino acid ordering based upon the reviewer's suggestion. We believe "'WFMLIVPYHAGSTDECNQRK'" is an improvement on the original suggestion, mainly due to the separation of negative- and positive-charged amino acids, the clustering of small amino acids, and the central location of tyrosine and histidine, which can both operate in some capacity as amphipathic residues. We note, however, that these

changes are somewhat subjective due to the difficulty in ordering amino acids in a linear fashion according to biophysical properties.

How are the property values for missing/gapped sequence positions handled? Are they assigned the mean value?

In AIMS, all missing or gapped sequence positions are left as a simple “0” in all matrices. When dealing with biophysical properties, this zero does not bias the analysis in a specific direction, as all property matrices are normalized to a mean of zero and a standard deviation of 1. In the calculation of mutual information, entropy, or frequencies, the gaps are accounted for as their own entry “-” so as not to bias amino acid frequencies. For instance, you can see in Figure S9 that the amino acid frequencies of rows 3-5 and 9-11 do not add to 1, because the gap is not included in this figure.

We note that the suggestion of reviewer 2 to add a “coverage” metric in AIMS helps to alleviate what may otherwise be misleading results in some instances of the standard analysis. Effectively suggesting to readers that results in these low-coverage regions should not be given much weight. Should users want to investigate certain low coverage regions, different alignment schemes can accomplish such a task.

The legend is messed up in Fig 4D

As mentioned in the response to reviewer 2, it appears that a rough draft version of Figure 4 (now 3) was uploaded upon resubmission. We apologize for the mistake, and thank the reviewer for pointing out the issue.

Figure 5A-- doesn't convey much information to me; does not seem to match the text description. "only P2 glutamine and isoleucine show up as distinct anchors for HLA-B*15 and HLA-A*02, respectively" Actually it looks like leucine and methionine have stronger relative preferences (darker blue) than isoleucine at P2.

We thank the reviewer for pointing out apparent inconsistencies between the figure and the text. We meant to refer to the C-terminal anchor (P14) in the case of isoleucine, not the anchor at P2. The text was misleading in this regard. We note, for this and the next comment regarding Figure 5 (now 4), that a metadata issue was discovered in the original analysis, which has been corrected in this version of the manuscript. As such, all panels of Figure 5 (now 4) have been altered, as well as some of the conclusions in the text. The interpretations remain largely the same with some differences in the magnitude of the effects. We have further included % enrichments of each amino acid to more directly tie together the text and the figure.

Figure 5B-- the handling of gaps makes this a very confusing plot; shouldn't the anchor positions be the lowest entropy positions? Instead we have these artificial 0 values suggestive of high conservation but they are just positions without any amino acids. Or worse, intermediate but still very low entropy values (pos 5) from a mix of gaps and non-gaps. This same issue extends to 5C where we have the most striking feature of the

plot coming from the difference along the diagonal (ie, just entropy) at this silly position 5 where one set of peptides has all gaps and the other doesn't.

The gap-induced features of Figure 5B (and in turn 5C) (now 4B and 4C) are resolved with the addition of a bit more context. First, as per the suggestion of Reviewer 2, we have added the coverage at each position to Figure 5B (now 4B) to alleviate the artifacts introduced into the data by sparse coverage in certain regions. This coverage applies to Figure 5 (now 4) panels A, B, and C, providing context to the location of gapped regions. These gapped regions are artificial, but important for providing context to the data. The combination of low coverage for the influenza peptides yet non-zero entropy tells you that the Influenza-derived peptides in this dataset have a less uniform length than the Ebola-derived peptides. While this does alter the mutual information figure, again with the context of the coverage, users can identify these biases in their data.

Figure 6: "the motifs identified by AIMS via the clustering in panel C" How, specifically, are motifs identified in the AIMS clusters? I couldn't find a description in the methods. A potential advantage of the GLIPH motifs is that they are nominally statistically significant based on a background repertoire. The AIMS clustering/motif ID procedure will presumably find clusters and motifs, even in TCRs from naive T cells without shared binding specificity. For example in shared V or J sequence regions.

Normally in the AIMS analysis, “motifs” are not specifically identified. The point of the comparison in the text and in panel 6D (now 5D) is to show that combinations of motifs such as SIR, SIRS, and IRS are entirely degenerate and provide no added information. AIMS instead utilizes biophysically similar clusters, which should take the place of motifs. The AIMS motifs in panel 6D (now 5D) are taken directly from clusters and combinations of clusters for a direct comparison between AIMS and GLIPH.

Specifically, the motifs were identified using a sequence logo plot and then converting regions of biophysical similarity to an “X”. So, in the case of motif RSXY which corresponds to clusters 4 and 5, we had sequences containing RSSY, RSGY, and RSAY**,** which are identical motifs save for the small polar residues which are replaced by an “X” in the final motif. In other words, for these figures the motifs were identified by hand for easy comparison to GLIPH, but the standard AIMS analysis makes it easy to identify sequence similarities in clusters of biophysically similar TCRs.

Regarding the identification of statistically significant clusters, AIMS is also capable of identifying statistically significant enrichments of certain metadata groups by comparison of cluster groups to randomly permuted groups of sequences. Reviewer 4 requested a test with simulated data, so such a statistical consideration is shown in Review Figure 1 for this test data. We find statistically significant enrichments in certain clusters (black bars) but not others (white space) helping AIMS users identify what may be spurious or random clusters and others which are statistically significant.

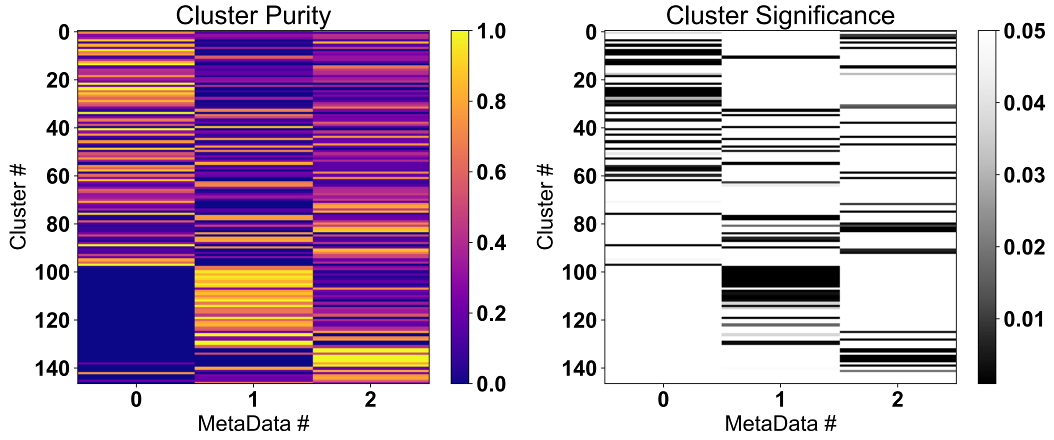

Review Figure 1: Clusters are generated from a pool of three simulated datasets (discussed in more detail below) and assessed for purity and significance with respect to membership of each biophysically distinct simulated dataset. (Left) Cluster purity is measured simply as (Number of sequences from a dataset) / (Number of sequences in cluster). Each dataset is given a simple metadata # (1 – Negative charge dataset, 2 – Positive charge dataset, 3 – random sequence dataset). (Right) The associated significance of these clusters given as p-values. White space gives clusters with p > 0.05, grey or black has p < 0.05. Significant clusters mostly overlap with clusters with near 100% purity.

However, this does not solve the issue raised by the reviewer regarding an analysis of naïve T cells. Without some comparison group, the permutation test will not work to identify statistically significant clusters. AIMS does, however, have an entropy-weighting feature to prevent clustering due to shared V- and J- segments, as seen in Review Figure 2. This feature is critical for large datasets (over 10,000 total receptors), as seen below. This entropy-weighting ensures that even in naïve repertoires, only biophysically similar receptors will be clustered, with an emphasis on the central peptide-contacting regions of the TCR. Such corrections ensure the clustering of biophysically similar TCRs, which regardless of antigen experience are meaningful clusters. To our knowledge, such analysis of naïve repertoires is not meaningful in GLIPH.

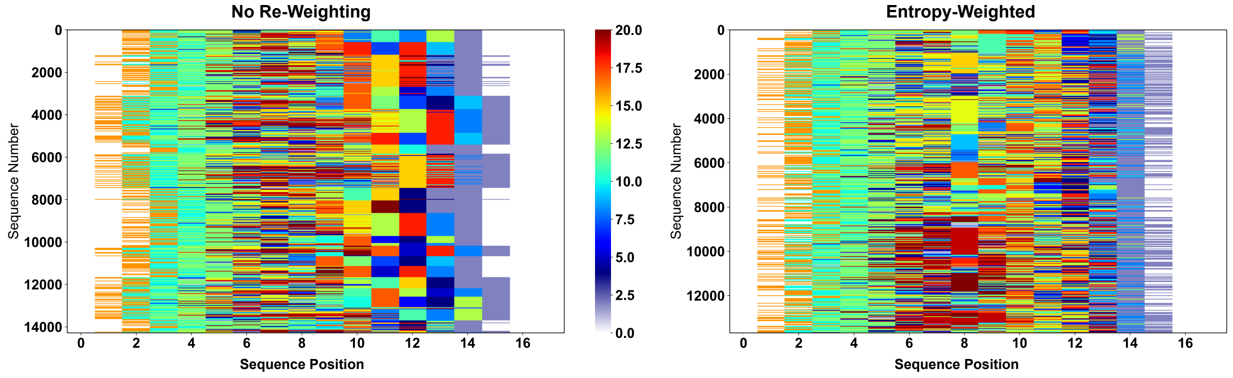

Figure 2: Comparison of the effects of standard AIMS clustering (left) and entropy-weighted clustering (right) carried out on the same dataset (simulated data discussed below). Notice that the similarities in the clusters (lines between clusters omitted for clarity) are solely in the TRBJ-encoded region in the standard clustering, but shifts to the center of the TCR for the entropy-weighted clustering. Not only are central residues picked up by this entropy-weighting, but the surrounding residues tend to be enriched in specific biophysical properties (notice the enrichment in red in the lower third of the figure, indicative of positive charge).

Reviewer #4: This manuscript describes a novel analysis tool for the characterization of immune-related peptides/proteins based on their amino acid sequence. The authors have expanded an analysis pipeline that was previously developed only for antibody repertoire characterization, to now process and characterize essentially any set of amino acid sequences, with a focus on immune-related molecules. To achieve this generalized functionality, they apply sophisticated alignment, encoding and clustering approaches.

The analysis of immune-related peptide/protein repertoires is becoming increasingly important with a fast growing number of available datasets. Understanding the diversity and functional properties of peptide/TCR/antibody repertoires is a key requirement for antigen-specific immunotherapy approaches. The aim of this manuscript is therefore of great importance and very timely.

We thank the reviewer for this concise summary highlighting the novelty of the work presented in this manuscript.

However, while the goal to characterize any ‚immune repertoire' is applaudable, it comes with the significant risk to ignore or neglect specific characteristics of certain repertoire types (e.g. peptide vs. protein, MHC vs. TCR). In fact, it remains unclear to me what the specific questions would be that could be addressed with such a generalized tool that can purportedly analyze all sorts of sequences.

We agree with the reviewer that the individual characteristics of each repertoire type is critical to the proper analysis of these molecules. This is why in the deployment of the software, there are specific "modes" for each type of analysis (see supplemental Figure S3). The processing of each repertoire type is distinct (i.e. a TCR/antibody mode, an MHC mode, and a multi-sequence alignment mode). This processing generates a unique encoding of amino acid sequences into matrices containing the key structural features of each specific molecule, and from there all of the downstream analysis follows an identical pipeline (as highlighted in Figure S2).

The AIMS software has been successfully applied to provide biological insights to the individual analysis of antibodies (Boughter et al. *eLife* 2020), TCRs (work in review), MHC molecules (work in review), SARs-CoV-2 RBD regions (Jiang et al. *Comm. Bio.* 2023), and even the non-immune related molecular Dpr-DIP interactome (Nandigrami et al. *J. Phys. Chem* B 2022). In each of these cases, unique AIMS encodings were developed for analysis of these individual molecular species. The AIMS "architecture" that is identical across repertoires is only applied after this initial encoding, such that the key details for each unique receptor highlighted by the reviewer are not lost. The application of AIMS to multiple molecular species simultaneously will be addressed in later responses to the reviewer.

From the abstract and main text it sounds as if repertoires of interacting peptides and MHC molecules could be magically clustered in order to identify certain peptides bound by certain MHC molecules. However, an MHC molecule doesn't bind a peptide because

it has a similar sequence, so I don't understand how this should work. The same is true for the interaction of TCR (CDR3) and MHC:peptide complexes, how would the clustering based on amino acid sequence help in understanding their interaction?

While it is certainly true that a simple concatenation of MHC and peptide sequences into a single matrix and subsequent clustering wouldn't generate any meaningful results, the simultaneous analysis framework of AIMS makes it possible to identify matching patterns in each repertoire. We can look at an example of such careful analysis by considering the interactions between peptides and the canonical binding pockets on MHC molecules. To do this, we generate a new encoding strategy (as discussed above) for MHC anchor regions and highlight the ability of AIMS to identify (previously discovered) anchor preferences. Review Figure 3 shows how the AIMS analysis can be used to distinguish between binders and non-binders for specific peptides isolated from the immune epitope database.

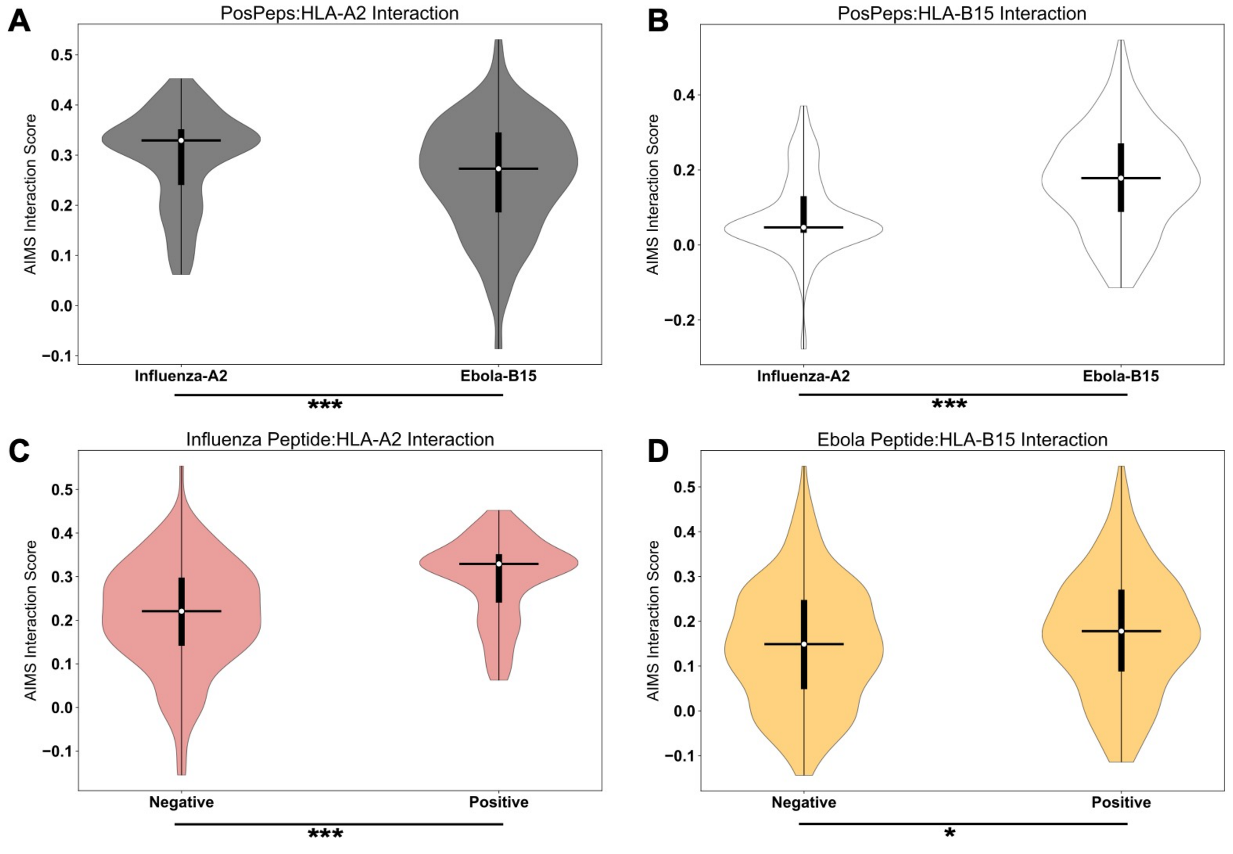

*Review Figure 3: A demonstration of the utility of cross-repertoire analysis using the AIMS interaction score function. These interaction scores are calculated across 1000 peptides and two MHC molecules (either HLA-A*02:01 or HLA-B*15:01). Violin plots show the distribution of these scores while thick vertical black lines give the quartiles and thin horizontal black lines with a white dot in the center give the medians for each distribution. When comparing known binders of HLA-A2 (Influenza-A2) and HLA-B15 (Ebola-B15) we see that their allele specificity is evident from the interaction scores, with the peptides having higher interaction scores with their respective proper interaction partners (Influenza-A2 peptides with HLA-A2 (A), and Ebola-B15 peptides with HLA-B15 (B)). Analyses within each individual dataset (Influenza-A2 peptides with HLA-A2 (C), and Ebola-B15 peptides with HLA-B15 (D)) show that binders and non-binders can likewise be distinguished using the AIMS interaction scores. Statistical significance of differences assessed using a permutation test comparison of medians, * $p < 0.01$, *** $p < 0.0001$.*

Interaction scores are based upon the AIMS-encoded matrices and the biophysical properties of the amino acids at each encoded position (See Supplemental Table 3 for the interaction matrix). Scores are reported as single values averaged across the interaction scores at each individual site. Amino acids that make up each MHC pocket are identified according to the interaction matrix of Nielson et al. [*Plos ONE* 2007 “NetMHCPan”] and Zhang et al. [*Bioinf* 2009 “PickPocket”]. Unlike these programs (NetMHC and PickPocket), however, the AIMS interaction scores undergo no training to identify binders and non-binders. An accounting of the fundamental interactions between amino acids within the AIMS architecture is sufficient to generate the statistically significant differences shown in Review Figure 3. It is important to note, however, that this example was provided as a test case to provide examples of the utility of cross-repertoire analysis. For AIMS to properly function as a tool for identifying binding and non-binding peptides reliably, more work would be necessary.

We further note that AIMS has been utilized for a more novel characterization of protein-protein interactions, namely via the quantification of the interaction propensity between the germline regions of TCRs and the alpha-helices of MHC (Boughter et al. *BiorXiv* 2023). More details on how these interaction scores are calculated can be found in the methods section of this cited preprint.

As the authors point out, there is already excellent software to characterize each of the different immune repertoire types (antigen/TCRs/MHCs/antibodies). I’m lacking the imagination in which circumstances I would need a tool that can characterize all in one go (and what I could learn from this). The authors state for instance that “Software that compares, for instance, peptide and TCR repertoires typically give a simple binary “yes” or “no” to questions of binding, making the identification of trends within or across these repertoires difficult”. But what kind of trend would I expect across these repertoires? It would be fantastic if this tool could predict which TCR can bind which peptide, but this would be wishful thinking, as this has nothing to do with amino acid sequence similarity between peptide and TCR.

First, we would like to point out that while amino acid similarity has nothing to do with the prediction of TCR-peptide interactions, amino acid complementarity has everything to do with these interactions. So, one of our next steps is to generate such a “complementarity score” to go along with the aforementioned interaction score. We have already done so for TCR-MHC germline interactions (as highlighted above) using the AIMS architecture and hope to work towards doing the same for peptides. However, looking at crystal structures of TCR-peptide-MHC complexes, it becomes clear that the problem is more complicated than simple complementarity of amino acids between TCR and peptide. There is an interplay of the entire TCR-peptide-MHC ternary complex, which may potentially be systematically deconstructed using multiple-receptor analysis as outlined in the present work.

Which brings us to the second point. AIMS is not simply for cross-receptor analysis [See the “Going Beyond Receptor Clustering & Motif Analysis” Section]. It is also for single-repertoire characterization. While other types of repertoire analysis software are great

for identifying clusters of receptors, their analysis typically ends at this stage. AIMS goes further, characterizing the individual clusters (or entire repertoires) and generating quantifications of each identified group. Boiling the software down to its most ambitious goals leaves out perhaps its best feature, which is an entirely unique approach to repertoire characterization not attempted in any amino acid sequence analysis prior.

Another example is this statement: “[AIMS] allows for cross-receptor analysis and the identification of patterns in the corresponding trends of interacting molecules.” I have no idea what the authors mean with this (‘patterns in the corresponding trends of interacting molecules’).

By “patterns in the corresponding trends of interacting molecules” we mean situations such as the above discussed case of the anchor regions of MHC molecules. So, for example, a cluster of peptides may show an enrichment for a basic residue at the C-terminus. It has been shown that A03 HLA supertype has a preference for basic residues in the F’ pocket, while no other supertypes have this preference [Sidney et al. *BMC Immunology* 2008]. AIMS clustering of MHC molecules by their pocket residues would likely align closely with these supertype designations, corresponding to a “trend” of certain biophysical properties. This complementary “trend” would identify interacting molecules, i.e. A03 HLA supertype members and those peptides with basic residues at the C-terminus. The AIMS biophysical property analysis, again going beyond simple clustering of molecules, aids in the identification of these trends.

I’m also concerned by the issue, which became particularly clear through the highly insightful review by reviewer #2, that for many of the possible parameter settings that impact on the analysis outcome are not well described. The authors brush over this by stating that the user should know what they are doing and should try many different settings to see how it affects their results. But what is the user to do if the results change depending on the setting? How are they to know which are the ‘right’ results (and settings). I would argue that the developers of a tool should be the ones to show the user which parameter settings are critical for appropriate analyses and give guidelines what should be used in which case. The authors state that the tool should be used for exploration, but the user needs some guidelines about how different results are to be interpreted, based on thorough testing of known/simulated data.

We agree with the reviewer that a thorough, controlled testing of the software with simulated data may greatly improve the confidence of users in the functionality of the software. We have added the option to simulate data into AIMS (details added to the Methods), and present the results as Supplementary Table S2, in addition to the previously generated tests of the effects of encoding-alignment on analysis. We again find limited effects on the choice of alignment, clustering algorithm, and projection algorithm in the overall statistics, but note that there are nuanced differences in the types of key receptors and regions of interest identified with each mode of analysis that are not captured by these tables. For instance, while the “Left” alignment scheme appears to perform somewhat comparably to the “Standard” analysis, the clusters are

strongly determined in part by J-gene usage in the “Left” alignment, even with the entropy re-weighting discussed above. However, such insights may be desirable in some contexts, for example, researchers may be interested in biophysically similar antibodies or TCRs utilizing a specific V- and J-gene segment (IGHV1-69, for instance, has tendencies towards broad neutralization). As such, it is difficult to call such a feature “wrong”.

Further, regarding the responsibilities of developers, we would argue that using any software without an understanding of the underlying assumptions of that software represents negligence on the part of the user. For instance, the use of the analysis software Seurat has been a boon to the field of single cell RNA sequencing, but using this software without an understanding of the normalization features, the effects of dropout in RNAseq analysis, and the strengths and weaknesses of RNAseq as a technique in general may generate misleading results.

We do, however, believe that improvements to the software can reduce the burden placed on users. To this end, and strongly motivated by reviewer comments for this manuscript, we have included simulated data, warnings and visualizations of low coverage areas, and direct testing of the impact of different alignment strategies on data analysis. We believe these additions have greatly improved the overall usability of AIMS.

Beyond the relevance and usability of the new tool, the manuscript describes in great detail the computational steps performed by their pipeline. I have to admit that I lack the computational and mathematical background to judge some of the employed approaches, so I’m not questioning those. However, reviewer #2 seems to have dipped deeply into the methodology and examined/reviewed it carefully, finding it generally sound.

We hope that the newest version of the text, with alterations as suggested by Reviewer 2, is more readable than previous drafts.

The comparison to existing tools (e.g. GLIPH or TCRdist for TCR clustering) is only partly helpful, since it is difficult to interpret the differences to their results. Which tool is right when there are differences? Here it would be helpful to have a simulated test dataset where we know what to expect in terms of output.

It should be noted, that, of course, AIMS can be used as a complementary piece to the existing tools GLIPH and TCRdist. To our knowledge, both of these software packages have limited utility in the downstream characterization of identified clusters, which AIMS was originally primarily designed to do. Users have the option to cluster TCRs (although not antibodies, MHC, or peptides) in GLIPH or TCRdist and use the characterization features of AIMS after importing these clustered receptors.

Additionally, as mentioned above, we have generated such a simulated dataset as suggested by the reviewer. To directly compare the performance of GLIPH, TCRdist,

and AIMS for TCR clustering, we simulate three repertoires with distinct biophysical properties in the center of CDR3B (see updated Methods for more details). We find that of the three, GLIPH performs by far the worst in terms of cluster purity and number of receptors clustered (Supplemental Table S2). However, as mentioned above, the use of simulated data may represent an unfair test of GLIPH. If assessing performance solely on cluster purity, TCRdist performs the best of the three algorithms. However, it is important to note that the AIMS distance metric performs just as well, suggesting distance generally is the metric of choice for generating "pure" clusters. Further, if we look at the AIMS clusters (Review Figure 4), we note that "impurities" in the charged clusters largely come from the "random" dataset, identifying randomly generated sequences with charge patterns matching the specifically generated charged sequences. Such tolerance may in fact be important when looking for things like cross-reactive receptors, suggesting yet again that there does not exist any "right" answer to the problem of receptor clustering.

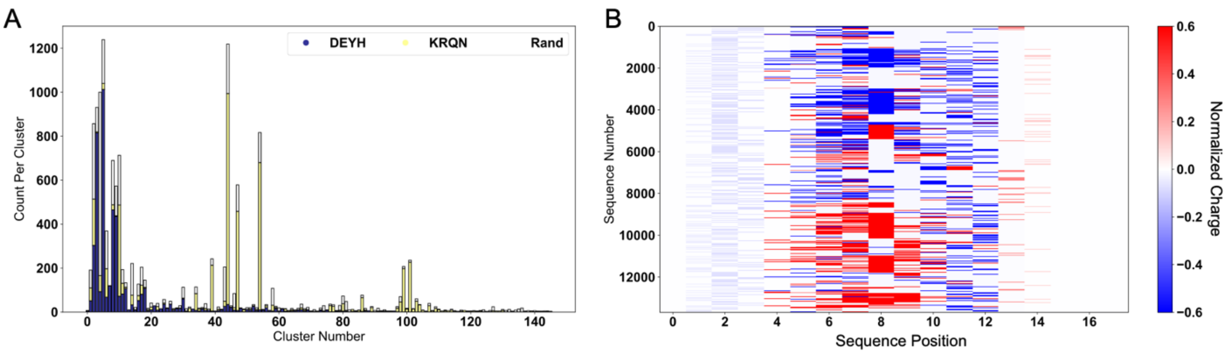

Figure 4: Clustering of simulated data using the standard AIMS analysis pipeline identifies moderately "Impure" clusters that are nonetheless biophysically similar. (A) Cluster membership of each identified cluster visualized using stacked bar plots. The majority of clusters are overwhelmingly comprised of one of the three simulated datasets, with impurities largely coming from the randomly generated sequences (white). (B) Visualization of the clustered sequences colored by the normalized charge of each sequence. As should be clear from the figure, positively charged sequences occur within the first 20 clusters (first 6000 sequences), while negatively charged sequences dominate clusters 40 and on (sequences 6000-12000). Lines separating the clusters are omitted for clarity, due to the large number of small clusters.

Overall, it seems to me that the authors are here describing an advanced amino acid sequence clustering tool that might be useful also in the context of immune repertoire analysis, e.g. for the analysis of a TCR sequence dataset. If it does better in this clustering than other existing tools remains unclear to me. However, I find the current framing with cross-repertoire analyses highly confusing and even misleading.

We should note that at no point in this manuscript do we suggest that cross-repertoire analysis would take the form of a simple clustering of combined datasets (say TCR and peptide) to generate conclusions about specificity. Cross-repertoire analysis looks more like the examples discussed in the earlier response to Reviewer 4, in the discussion of peptide-MHC pocket interactions. The analysis we perform goes beyond simple clustering. See sections "Going Beyond Receptor Clustering and Motif Analysis", and "Generating Quantitative Metrics of Repertoire Diversity and Amino Acid Patterning".

What is the biological meaning of comparing amino acid sequences of TCRs with those of peptides?

We further note that comparisons between the amino acid sequences of TCRs with those of peptides are very important for understanding the biophysics of a T cell response to antigen. TCRs bind peptides presented by MHC to initiate an immune response [Meuer et al. *J. Exp. Med.* 1983, Haskins et al. *J. Exp. Med.* 1983], making comparison of the amino acids comprising these molecular species highly important.
